# Non-Equilibrium Thermodynamics of Heat Transport in Superlattices, Graded Systems, and Thermal Metamaterials with Defects

**DOI:** 10.3390/e25071091

**Published:** 2023-07-20

**Authors:** David Jou, Liliana Restuccia

**Affiliations:** 1 Grup de Fisíca Estadística, Universitat Autònoma de Barcelona, 08193 Bellaterra, Spain; david.jou.mirabent@gmail.com; 2 Institut d’Estudis Catalans, Carme, 47, 08001 Barcelona, Spain; 3 Department of Mathematical and Computer Sciences, Physical Sciences and Earth Sciences, University of Messina, Viale F. Stagno d’Alcontres, 31, 98166 Messina, Italy

**Keywords:** continuum thermodynamics, heat transport, non-equilibrium thermodynamics, superlattices, thermal metamaterials, graded systems

## Abstract

In this review, we discuss a nonequilibrium thermodynamic theory for heat transport in superlattices, graded systems, and thermal metamaterials with defects. The aim is to provide researchers in nonequilibrium thermodynamics as well as material scientists with a framework to consider in a systematic way several nonequilibrium questions about current developments, which are fostering new aims in heat transport, and the techniques for achieving them, for instance, defect engineering, dislocation engineering, stress engineering, phonon engineering, and nanoengineering. We also suggest some new applications in the particular case of mobile defects.

## 1. Introduction

The electrical, mechanical, and thermodynamical aspects of many kinds of materials are well-known and well-understood. In this review paper, we focus instead on the nonequilibrium thermodynamics and dynamics of anisotropic and inhomogeneous materials, with special interest on three kinds of systems: superlattices [1,2,3,4,5,6,7,8,9,10,11,12,13,14,15,16], graded systems [17,18,19,20,21,22,23,24], and thermal metamaterials [25,26,27,28,29,30,31,32,33,34,35,36,37,38,39,40], for which anisotropy and/or inhomogeneity have been shown to be essential to achieve some particular functionality. Such systems, which we describe below, are opening new perspectives on material sciences, either at macroscopic, mesoscopic or nanoscopic scales. We further consider the influence of defects (point defects or line defects) and of applied stress, both in the case that the defects are fixed and in the case they may move [41,42,43,44,45,46,47,48,49,50,51,52,53,54]. These topics have received a further impetus with the possibility of nanomanipulation of the systems by means of nanotechnology techniques; to describe heat transport in such nanosystems, one must go beyond Fourier’s law, which is a very active practical and theoretical topic [55,56,57,58,59,60,61,62,63,64,65,66,67,68,69].

From a physical point of view, these issues require a detailed formulation of the constitutive equations for anisotropic and inhomogeneous systems. In some aspects, the situation is conceptually simple: it is sufficient to consider the matricial character of thermal conductivity and other transport coefficients. However, determining the optimal mathematical values of the transport coefficients, and identifying or manufacturing the suitable materials to achieve those values, is a very challenging task. In some other cases, the generalization has additional difficulties, because the anisotropies and inhomogeneities allow some physical couplings between thermodynamic fluxes and forces which are not found in isotropic and homogeneous systems, which become basic for relevant applications.

Understanding the influence of anisotropy, heterogeneities, interfaces, and defects on transport properties (heat transport, electric transport, thermoelectric conversion, and so on) of superlattices, graded systems, and thermal metamaterials is an interesting topic for new and very active kinds of material engineering: defect engineering, dislocation engineering, stress engineering, phonon engineering, and nanoengineering (for references to these fields, see Section 2.3.1, Section 2.3.2, Section 2.3.3 and Section 2.3.4).All these fields aim towards suitable modulation of transport coefficients, allowing one to control in detail the several fluxes in physical systems, to improve thermal management and energy conversion and storage or for other aims.

Indeed, from a theoretical perspective, one may try to obtain the spatial distribution of temperature, electrical potential, and so on, optimizing some features of the system. For this, it is necessary to know the spatial distribution of the values of the transport coefficients. The aim of material sciences is to find particular materials, yielding the required values of the transport coefficients at the several positions. Thus, the physical understanding of transport laws, the mathematical solutions of the set of equations under given boundary conditions, in view of the required functionalities, and the manufacturing of the corresponding materials have in this field an intimate and interesting interplay.

The main purpose of this review is to bring to researchers in nonequilibrium thermodynamics the inspiration provided by these recent developments in heat transport engineering, and provide material engineers with a useful theoretical tool to describe in a systematic way a number of new effects arising in the mentioned systems (superlattices, graded materials, thermal metamaterials, point or line defects, materials under stress). In particular, we pay a special attention to the consequences of micro-structure, heterogeneities, anisotropy, and defects on the transport properties of the systems, mainly, on heat transfer and charge transfer. Then, we derive the form of constitutive equations and rate equations for heat and charge fluxes and defect field and interfaces.

A macroscopic nonequilibrium model for semiconductor crystals and superlattices with defects, formulated in previous papers [41,42,43,44,45,46,47], is enlarged, in the framework of rational extended irreversible thermodynamics with internal variables [55,56,57,58,59,60,61,62,63,64,65,66,67,68,69,70,71,72,73,74,75] (see also [76], where the same procedure is used), where the dissipation inequality is exploited using Liu’s procedure and additional rate equations for dissipative fluxes and the defects are given as ansatzes in the form of balance Equations (see (44) in Section 4 [70,71,72,73,74]).

In Section 2, we provide an overview over the mentioned new aspects of material engineering with emphasis on illustrations on thermal diodes and transistors, thermoelectric energy conversion, thermal cloak, and thermal intensification. In Section 3, several new functional aims in heat transport are presented. In Section 4, the basic laws are presented, while in Section 5, the constitutive theory and the rate equations for the fluxes and the defects are described. In Section 6, some new applications of the equations are suggested when the defects are mobile. In Section 7, a summary and outlook are provided.

## 2. Current Developments and Frontiers in Heat Transport

In principle, heat transport is a classical branch of sciences. Fourier’s law (1810) provided a conceptually elegant and practically useful mathematical basis for the description of heat conduction. With the advent of nanotechnology, the possibility of an accurate treatment of materials at nanoscale implied a new range of heat transport, because the fundamental laws (Fourier’s law, valid for diffusive transport) are no longer valid for ballistic transport, when the size of the structures or of the systems becomes comparable to the mean free path of heat carriers. This has led to a renewal of heat transport theory, namely its laws and its aims [55,56,57,58,59,60,61,62,63,64,65,66,67,68,69]. For instance, we may refer to phononics (control of phonons allowing for thermal diodes, transistors, and logical gates), metamaterials (allowing thermal invisibility, heat shielding, or heat intensification), and nanosystems (nanowires, thin layers, nanostructured materials, and microscopic phononic systems), allowing to incorporate the influence of the characteristic structure size [57,58,59,60].

Here, we provide a simple perspective of several problems, in a qualitative view, in order that the reader may have in mind the physical situations we are examining. We consider three geometrical aspects regarding the structure of the systems, namely superlattices (repetitive structures) [1,2,3,4,5,6,7,8,9,10,11,12,13,14,15,16], functionally graded materials (systems with an increase of some particular property along one direction) [17,18,19,20,21,22,23], and thermal metamaterials (artificially manufactured materials with controlled anisotropy and heterogeneity) [25,26,27,28,29,30,31,32,33,34,35,36,37,38]. We also include some material aspects related to the defects (point defects and line defects) and to the applied stress.

In material engineering, in the particular aspects we are considering in this review, one aims to control the structural and compositional aspects and the amount and type of defects and the applied stress in order to tailor the transport properties and dynamical behavior of the system. Of course, typical aspects of equilibrium thermodynamics are also taken into consideration, as for instance the phase diagram and transition temperatures, which are essential for the thermodynamic stability of alloys and multicomponent systems, but they are not of interest in the present paper, devoted to nonequilibrium aspects.

### 2.1. Simplified Illustrative Expression for the Thermal Conductivity

As a preliminary information we give a mathematically concrete form of the transport coefficients useful for illustration of our diverse considerations. We assume that the thermal conductivity (and, analogously, other transport coefficients) depends on *T*, on the composition, on the applied stress τ, and on the concentration of defects *c*, namely, λ(T,τ,c). Here, τ will usually denote the lateral stress acting on a system (or, in some cases, one could take as τ the square root of the second invariant of the tensor τ, i.e., $\tau \equiv {\left(\tau :\tau \right)}^{2}$, with “:” denoting the full contraction of the tensor.

In fact, in the systems we are discussing, the thermal conductivity is a tensor (expressing the anisotropy) and it depends on the position (expressing the inhomogeneity). For instance, in multilayered systems with the layers orthogonal to the *z* axis, the tensorial thermal conductivity is as follows:

(1) $$\lambda =\left|\left|\begin{array}{ccc} \lambda_{xx}\left(z\right) & \lambda_{yx}\left(z\right) & 0\\ \lambda_{xy}\left(z\right) & \lambda_{yy}\left(z\right) & 0\\ 0 & 0 & \lambda_{zz}\left(z\right) \end{array}\right|\right|.$$

For the sake of concreteness, we assume for the components $\lambda_{ij}$ of thermal conductivity tensor a simple expression of the following tentative form (we omit the subscripts for simplicity):

(2) $$\lambda \left(T,\tau ,c\right)=\frac{\lambda_{0}\left(T\right)}{1+\beta_{1}T^{a}c^{b}+\beta_{2}T^{d}{\left(\tau -\tau_{0}\right)}^{f}},$$

where $\lambda_{0}\left(T\right)$ is the thermal conductivity of the material without defects (c=0) and at a reference stress $\tau =\tau_{0})$, a,b,d,f are constant exponents, $\beta_{1}$ and $\beta_{2}$ constant coefficients of order unit (for instance, their indicative values are between 0.1 for small defects in a matrix of big atoms, and 10 for big defects in a matrix of small atoms), and $\tau_{0}$ is a reference value of the applied stress. These coefficients and exponents depend on the materials. Expression (2) means that defects and applied stress usually reduce the thermal conductivity.

Indeed, in the simplest relaxation-time form, the thermal conductivity is given by $\lambda =\left(1/3\right)\;c_{v}\;v_{0}^{2}\;\tau_{c}$, where $c_{v}$ is the specific heat per unit volume, $v_{0}^{2}$ is the average of the square velocity of heat carriers, and $\tau_{c}$ is the average collision time. Thus:

$$\frac{1}{\lambda }=\frac{3}{c_{v}\;v_{0}^{2}\;\tau_{c}},\;\mathrm{and}\;\mathrm{we}\;\mathrm{consider}\;\mathrm{that}\;\frac{1}{\tau_{c}}=\frac{1}{{\left(\tau_{c}\right)}_{0}}+\frac{1}{\tau_{def}}+\frac{1}{\tau_{s}},$$

where $\frac{1}{{\left(\tau_{c}\right)}_{0}}$ is the collision frequency at lateral stress $\tau =\tau_{0}$ and without defects, $\frac{1}{\tau_{def}}$ is the frequency of collisions with defects, and $\frac{1}{\tau_{s}}$ is the increment of frequency of collisions due to an extra applied stress. The former expression of ${\frac{1}{\tau }}_{c}$ is a particular case of the so-called Matthiessen’s rule, whose applicability to systems with defects has been studied, for instance, in [77,78]. It may arise if one considers that the frequencies of the several kinds of scattering may be considered as additive, treating the scattering events as independent.

We assume that $\frac{1}{\tau_{def}}=\beta_{1}\frac{T^{a}c^{b}}{{\left(\tau_{c}\right)}_{0}}$ and $\frac{1}{\tau_{s}}=\beta_{2}\frac{T^{d}{\left(\tau -\tau_{0}\right)}^{f}}{{\left(\tau_{c}\right)}_{0}}.$

Thus, we obtain:

(3) $$\frac{1}{\lambda (T,\tau ,c)}=\frac{1}{\lambda_{0}\left(T\right)}\left[1+\beta_{1}T^{a}c^{b}+\beta_{2}T^{d}{\left(\tau -\tau_{0}\right)}^{f}\right].$$

where the second term in the brackets describes the collisions of heat carriers with the defects, and the third one the increase (or decrease) of the collision rate, when an extra stress $\tau -\tau_{0}$ is applied to the system. The value of *c* is imposed by the manufacturing conditions, and τ is controlled by the forces applied on the boundaries.

To achieve a maximum simplicity, we consider that these effects are relatively small (in the case when *c* and $\left(\tau -\tau_{0}\right)$ are small and the coefficients $\beta_{1}$ and $\beta_{2}$ are small) and that (3) may be rewritten in the first-order approximation as follows:

(4) $$\lambda \left(T,\tau ,c\right)=\lambda_{0}\left(T\right)\left[1-\beta_{1}T^{a}c^{b}-\beta_{2}T^{d}{\left(\tau -\tau_{0}\right)}^{f}\right].$$

In fact, more complicated expressions yielding, for instance, a saturation of the decrease of λ for high values of *c* or $\tau -\tau_{0}$ should be used if *c* is high enough.

If the size of the system, for instance the transversal size or the longitudinal size, let us say *L*, is so reduced that it becomes of the order of the length of the mean-free path of the heat carriers, *l*, the transport properties also depend on $\frac{l}{L}$, the so-called Knudsen number. There are several sophisticated expressions for such dependence. The simplest one is as follows (see [61]):

(5) $$\lambda_{effcross-plane}\left(T,c,\tau ,\frac{l}{L}\right)=\frac{\lambda_{0}\left(T,c,\tau \right)}{1+a_{cp}\left(T,c,\tau \right)\frac{l}{L}},$$

where λ(T,c,τ) is the expression of the thermal conductivity for the bulk system (for instance, the forms (2) or (4) or more precise and sophisticated expressions) and the denominator provides the size-dependent correction, where $a_{cp}\left(T,c,\tau \right)$ is a numerical coefficient whose value depends on the form of the cross section of the system and on the roughness of the lateral walls of the system (the higher the roughness, the higher the reduction of the effective thermal conductivity). An alternative expression obtained from a higher-order asymptotic expansion of the transport equation for the heat flux is as follows (see [62]):

(6) $$\lambda \left(T,c,\tau ,\frac{l}{L}\right)=\frac{\lambda_{0}\left(T,c,\tau \right)}{2\pi^{2}}{\left(\frac{l}{L}\right)}^{2}\left[\sqrt{1+4\pi^{2}{\left(\frac{l}{L}\right)}^{2}}-1\right].$$

where $\frac{l}{L}\ll 1$, (5) and (6) reduce to the usual thermal conductivity and the heat flux is given by Fourier’s law, namely $q=\lambda_{0}\frac{∆T}{L}$. In the limit when $\frac{l}{L}\gg 1$, instead, $\lambda_{eff}$ becomes $\left(L/l\right)\left(\lambda_{0}/a_{cp}\right)$ and the heat flux becomes $q=(\lambda_{0}/la_{cp})∆T$, with ∆T being the difference of temperatures on the opposite borders of the system, instead of being proportional to the temperature gradient, $\frac{∆T}{L}$, as in Fourier’s law.

### 2.2. Three Current Frontiers in Functional Materials for Controlled Heat Transport

In this subsection, we describe three kinds of systems which are being developed to optimize some concrete functional requirements for heat transport. We refer to superlattices, graded materials, and metamaterials.

#### 2.2.1. Superlattices

**Figure 1:**
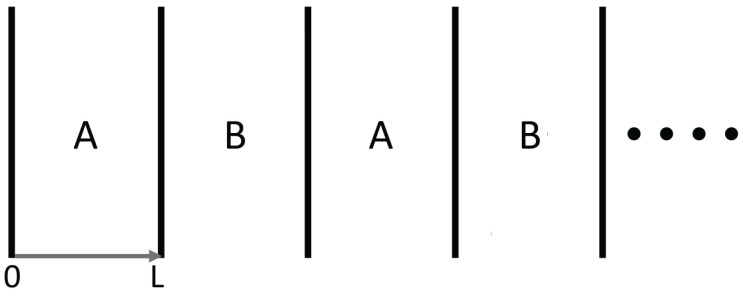
A superlattice of alternating layers of two semiconductors A and B. Note that, because of the geometrical structure, the system is anisotropic and its properties in the axis across the interfaces are not the same as in the axis parallel to the interfaces.

**Figure 2:**
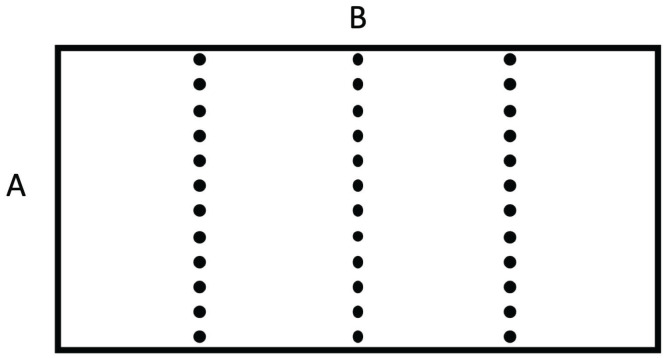
A superlattice composed of a regular distribution of point defects (B atoms or B nanodots, for instance Ge) into an originally homogeneous matrix of A, for example Si.

**Figure 3:**
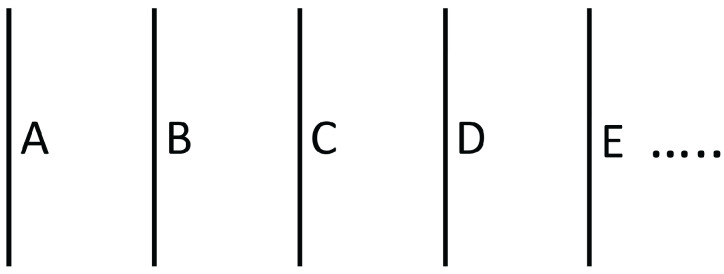
A graded system formed by a succession of different semiconductors.

**Figure 4:**
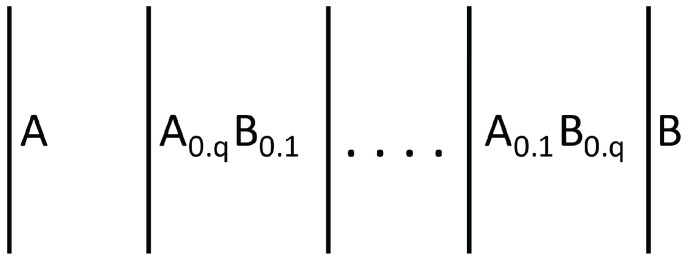
A graded alloy of semiconductors A and B with different compositions, $A_{x}B_{1-x}$, with *x* changing along one axis of the system.

Superlattices refer to systems physically structured in a periodic way, at a nanoscopic, mesoscopic or macroscopic scale [1,2,3,4,5,6,7,8,9,10,11,12,13,14,15,16,41]. For instance, in a number of applications, alternating layers of two different semiconductors, such as Si/Ge, $In_{x}Ga_{1-x}As/In_{x}Al_{1-x}As,$ and GaAs/GaAsP,…, have been considered. In more general terms, if we deal with two materials *A* and *B*, such as Si and Ge, two main kinds of superlattices are commonly considered (see Figure 1, Figure 2, Figure 3 and Figure 4): a series of parallel layers made of Ge and Si, alternatively, or a matrix of Si in which atoms of Ge (or groups of atoms of Ge) are periodically distributed (or alternatively, a matrix of Ge with periodically distributed atoms or groups of atoms of Si). In some occasions, small holes or pores may be periodically made perpendicularly to a material layer or regularly distributed in a volume. The atoms distributed in the matrix (or the interfaces between neighboring layers) contribute to the scattering of the heat carriers and have a strong influence on the transport properties of the system. In particular, they have a strong effect on the reduction of phonon thermal conductivity, but they do not reduce very much the electrical conductivity, because the mean-free path of the electrons is much smaller than that of the phonons. This reduction in thermal conductivity contributes to increasing the value of the figure of merit of the devices, thus enhancing the efficiency of thermoelectric energy conversion, as will be commented on in Section 3.3. Superlattices are also useful in increasing thermal insulation when this is required, for instance in the thermal shielding of spacecrafts for their fast reentry in the atmosphere.

The effective thermal conductivity along a superlattice must take into account the influence of the interfaces, which is usually accounted for through their corresponding thermal resistance, $R_{AB}$, defined as follows:

(7) $$T_{A}-T_{B}=R_{AB}q,$$

where $T_{A}$ and $T_{B}$ are the temperatures at each side of the interface. This expression is analogous to Ohm’s law $V_{1}-V_{2}=RI$, where *V* is the electrical potential, *R* is the resistance, and *I* is the intensity of the current along a wire or across a layer. The value of $R_{AB}$ depends on the materials A and B, on the thickness of the interface between the materials, and on the kind of interactions between phonons and the interface, and it is an active topic of research (see [79]).

On the other side, the thermal resistance of a layer of thickness *L*, with its external faces at temperatures $T_{A}$ and $T_{B}$, is $\frac{L}{\lambda }$; this comes from Fourier’s law q=−λ∇T, which when expressed as $q=\lambda \frac{T_{A}-T_{B}}{L}$ leads to:

(8) $$T_{A}-T_{B}=\frac{L}{\lambda }q.$$

In current technology, nanostructures have become very important, in such a way that *L*, the thickness of the layers, may be comparable to or smaller than *l*, the mean free path of free carriers. In this case, the contribution $\frac{l}{L}$ in the denominator of (5) becomes relevant and produces an additional increase of thermal resistance of the layer.

The total thermal resistance of the superlattice in the direction perpendicular to the interfaces is then the sum of the several thermal resistances of the layers and interfaces:

(9) $$\frac{L_{tot}}{\lambda_{effcross-plane}}=\sum_{i}\frac{L_{Ai}}{\lambda_{effA}\left(\frac{l}{L_{i}}\right)}+\sum_{j}\frac{L_{Bj}}{\lambda_{effB}\left(\frac{l}{L_{j}}\right)}+\sum_{k}R_{ABk},$$

which are obtained by adding the thermal resistances in series that we have seen above. Here, $\lambda_{eff\;A}\left(\frac{l}{L_{i}}\right)$ and $\lambda_{eff\;B}\left(\frac{l}{L_{j}}\right)$ refer to expressions (5) or (6) or any other expression for λ of materials A and B as a function of the Knudsen number.

The thermal conductivity parallel to the layers (in-plane conductivity) is given by an expression analogous to (5), but with a different value of the coefficient $\frac{l}{L}$ of the term at the denominator,

$$\lambda_{effin\;plane}=\frac{\lambda_{0}\left(T,c\right)}{1+a_{ip}\left(T,c\right)\frac{l}{L}}.$$

Furthermore, in this case, the resistances are in parallel. Thus, the resistances are not added but the reciprocal of the resistances, namely, the conductances. Thus, for the effective thermal conductance of the superlattices in the direction parallel to the interfaces we have, in contrast to (9),

$$\frac{\lambda_{eff\;in\;plane}}{L_{tot}}=\sum_{i}\frac{\lambda_{ieff}}{L_{i}}.$$

In this case, the thermal resistance of interfaces does not appear, because the heat flux is parallel to the interfaces.

#### 2.2.2. Functionally Graded Materials

Functionally graded materials are inhomogeneous systems, along which the material composition changes in such a way that some coefficients (or some combinations of coefficients) change in a given way required to optimize some function [17,18,19,20,21,22,23,24]. Particularly relevant situations are found, for instance, in the global optimization of thermoelectric energy conversion (see Section 3.3) and in metamaterials (see Section 2.2.3) in order to guide the heat flux along a given prescribed path. The materials may change in a discontinuous way (having for instance consecutive layers of different materials) or may consist of an alloy of composition $A_{1-x}B_{x}$, with the atomic fraction *x* changing continuously along the system, in some well-defined form as a function of the position. The transport coefficients depend on the composition *x*, so that controlling *x* allows one to control the thermal conductivity, between some range of values. The physical and engineering problem is to find the function x(z) optimizing some global behavior of the system. In fact, if a superlattice as discussed in Section 2.2.1 has not a regular repetition of the thickness of the layers $L_{A}$ and $L_{B}$, but $L_{A}$ and $L_{B}$ gradually increase along the system, the superlattice may also be considered as a graded system.

#### 2.2.3. Thermal Metamaterials

Metamaterials are particular combinations of materials, relatively sophisticated, leading to very exceptional properties with highly anisotropic and inhomogeneous thermal conductivity [25,26,27,28,29,30,31,32,33,34,35,36,37,38,39,40]. In some occasions, the metamaterial is based on a combination of concentric layers of materials with different thermal conductivities, so that the local thermal conductivity changes in a well specified way as a function of the radial position (namely, a radially graded superlattice). In other occasions, such metamaterials may be achieved by means of grooving thermally insulating materials and filling the grooves with a good thermal conductor and putting a succession of such layers slightly rotated with respect the precedent one or by rotating squares with auxetic property. The sophistication in the design and manufacturing of these systems allows one to control several functionalities of this material.

The source of inspiration for a number of metamaterials has come from previous developments in optical or dielectric metamaterials. In turn, such developments were based on mathematical progress about coordinate-transformation properties of the basic equations for the materials.

Metamaterials combine materials with very special nanostructure or mesostructure [25,26,27,28,29,30,31,32,33,34,35,36,37,38,39,40]. In particular, a suitable combination of concentric cylindrical layers of materials with different values of thermal conductivity may make that the heat flux in a cylinder between two parallel planes at different temperature flows tangentially to the cylinder without penetrating into it. In this way, one may protect from changes in the heat flux the systems placed inside the cylinder (thermal cloak). In some other devices, this cloaking function may be turned to a concentration function, focusing heat flux in a small central region instead of shielding it from the heat flux [25]. These functions will be discussed in more detail in Section 3.4.

In electrical transport, the optimization of electrical batteries is a challenge of crucial economical interest in renewable energies, electric cars, and portable electronic devices. It is necessary to reduce the charging time of the battery (implying an increase of electrical conductivity of the system), to enhance its electrical capacity (in order to store a bigger amount of energy), and to reduce resistive heating, so that electrical and thermal coefficients must be carefully controlled so that fast charging does not produce a dangerous increase in battery temperature.

### 2.3. Some Engineering Strategies for the Control of Transport Coefficients

To manufacture the kinds of systems mentioned in Section 2, a number of techniques have been developed, which have yielded the bases for new specializations of material engineering. In this subsection, we briefly explain four kinds of them.

#### 2.3.1. Defect Engineering

Defect engineering aims at finding the particular effects of the different kinds of defects on the several transport coefficients or on many other aspects of the system, as for instance in activating molecules for autocatalytic or electrocatalytic reactions, by introducing highly active sites for chemisorption and absorption, or for improving the efficiency of photovoltaic solar cells [80,81,82,83,84,85,86,87,88,89,90,91].

Defects may be zero-dimensional (point defects), one-dimensional (line defects, as for instance dislocations, which may form a complex network), two-dimensional (surface-like defects, as domain walls or phase boundaries), and three-dimensional (volumetric inclusions). All their equilibrium and nonequilibrium aspects are of much interest, in particular those arising from the structural differences between defects and the system. To reduce the thermal conductivity, vacancies and substitutions with big mass and size mismatch with respect to the atomic mass and size of the original material, implying a big lattice distortion, are especially useful, because they have larger effects on phonon collisions.

In some occasions, it is important that defect movement is avoided, whereas for some other applications, it may be of interest that they are able to move. This may be achieved in a more efficient way if the defects are electrically charged, because it is easier to produce forces on them from the outside. For all these topics, it is important to understand how the several parameters of the defects (concentration, location, chemical nature, geometric characteristics, crystal symmetry, etc.) influence the whole system and contribute to the effect being sought for. It is also important to know how to improve the introduction of defects into the original material. For instance, there are techniques such as ion implantation with high speed ion beams, or acid attack at the surface, that can degrade the walls and enhance their roughness.

#### 2.3.2. Dislocation Engineering

Dislocation engineering is a particular case of defect engineering. The dislocation lines disturb the periodicity of the crystal lattice. The effect of dislocations on thermal conductivity and other transport coefficients (electrical conductivity, Seebeck coefficient) has been well studied from experimental and theoretical points of view [1,2,3,4,5,6,7]. In fact, the dislocation density $\rho_{D}$ (total length of dislocation lines per unit volume, which has units of (length)${}^{-2}$) has only a minor effect on the thermal conductivity for dislocation densities smaller than a characteristic value dependent on the material and temperature. For higher values, there is a steep decrease of thermal conductivity. For instance, the critical dislocation density for Si and Ge is of the order of $10^{8}$ cm${}^{-2}$. This is due to phonon-dislocation scattering, which is negligible as compared to phonon-phonon scattering (for small dislocation densities $\rho_{D}$), but which becomes dominant for high values of $\rho_{D}$.

Furthermore, dislocations reduce electrical conductivity but, in contrast, increase the Seebeck thermoelectric coefficient in some range of dislocation densities (10${}^{6}$ cm${}^{-2}-10^{10}$ cm${}^{-2}$) due to an increase in the entropy of the carriers. Thus, for some ranges of dislocation density, the efficiency of thermoelectric energy conversion may be raised by dislocations, especially in low-dimensional structures (films, wires, or dots). This makes that dislocation engineering is becoming increasingly useful in the optimization of some aspects of semiconductor devices [1,2,3,4,5,6,7].

Dislocations have also a marked influence on mechanical properties. Not only do they modify the linear elastic coefficients, they also yield nonlinear elasticity, and irreversible plastic effects. Fixing the dislocations in some positions may reduce their contribution to the plastic behavior.

#### 2.3.3. Stress Engineering

The value of the transport coefficients of some materials may depend on the stress acting on them because such stress may modify the effective elastic constants of the microscopic lattice interactions. Since the stress may be changed from the outside in a way much simpler than the concentration of defects, stress engineering could be useful to modify in a relatively fast and reversible form the values of the transport coefficients. For instance, this may allow to regulate the rectification power or the amplification power of thermal diodes and transistors, or to adapt a thermoelectric energy converter optimized at a given temperature difference to external temperature changes [92,93,94,95,96,97].

#### 2.3.4. Phonon Engineering

The control of phonon flow is more difficult than that of charged particles, because they lack the possibility of being acted through electric and magnetic fields. However, other means of control have been devised and developed, by using the wide diversity of phonon wavelengths, and the corresponding diversity in mean free paths, depending on the kind of scattering mechanism (elastic and resistive phonon-phonon scattering, impurity scattering, and wall scattering) [57,58,98,99,100,101,102]. One may regulate these mechanisms with impurities, or in small systems of size comparable to the phonon mean path, or with the geometrical form of the boundary conditions, as for instance in the so-called phononic crystals. These systems consist of a regular arrangement of small holes; depending on the size and separation of such holes, some ranges of wavelengths may be forbidden to propagate. Another way to controlling phonons is by means of barriers at material interfaces. Phonon engineering aims at mastering these different possibilities of control. The equations describing those effects go beyond Fourier’s law and must incorporate nonlocal terms. Indeed, Fourier’s law is valid in the context of diffusive heat transport, whereas the phenomenology at small sizes (high values of Knudsen number) becomes much richer, incorporating phonon hydrodynamics and ballistic phonon flow.

## 3. Some Illustrations of New Functional Aims in Heat Transport

Material sciences aim to design and manufacture materials and devices improving or optimizing some particular function required by some technological scientifical purposes. They may aim to improve elastic, thermal, optical, magnetic, and electric properties. Since here we are dealing with nonequilibrium aspects, the most relevant aim of material engineering in this field is the control of transport coefficients (thermal conductivity, diffusivity, electrical conductivity, Seebeck and Peltier thermoelastic coefficients, electromagnetic absorption and emission, and so on). These coefficients may be controlled by means of material composition, stress, or structure [103].

These aims are more or less classical (as the increase of efficiency of thermoelectric energy conversion), whereas other aims are very recent and sophisticated, as for instance, the manufacturing of phononic devices (thermal diodes, transistors, and logical gates), thermal cloaks and thermal concentrators. In this review, we focus our attention on them [103,104,105,106,107,108,109,110,111,112,113,114].

New extreme requirements and new highly demanding tasks are another stimulus to new developments in heat transport. For instance, a high thermal conductivity is convenient when one must remove the heat of some systems, as for instance supercomputers, where heat production is very high and becomes a limiting factor for more advanced computers, whereas miniaturization reduces the thermal conductivity and increases dissipation density. In some other occasions, in contrast, a very low thermal conductivity is required, as for instance in the thermal sheltering of spacecrafts in planetary reentry. Achieving suitable ranges of thermal conductivities is also important in many engineering areas, as in the food technology devices, building insulation, efficient energy management, and environment protection.

One must always keep in mind a global view of the phenomena influencing the problem. For instance, to reduce the thermal conductivity of window panes by means of added defects, in such a way to enhance the efficiency of thermal insulation, one must consider the consequences of defects not only on thermal conductivity, but also on the optical properties of the material, in order do not reduce the optical transparency of the pane, or trying to enhance the reflectivity of relatively long wave infrared radiation (influencing the loss of energy radiation of the building) without reducing the transparency to relatively short wave infrared radiation, providing warming with solar radiation.

Here, we illustrate some particular situations through very simplified models, using the simple expression (3) for $\lambda (T,c,\tau ,\frac{l}{L})$, allowing the reader to understand without much effort the essential concepts and grasp their potentialities. Truly realistic problems require much more complicated expressions for the thermal conductivity and massive computational effort. We deal with the behavior of thermal conductivity and its applications to heat rectification and to thermal transistors, with the regulation of thermal and electrical conductivities and Seebeck thermoelectric coefficient to enhance the efficiency of thermoelectric energy conversion or of refrigeration power in devices with an inhomogeneous temperature distribution, and with the use of anisotropy to guide heat flow through annular regions of a material inserted into a homogeneous material and achieve a thermal cloak or a thermal condenser.

### 3.1. Heat Rectification and Thermal Diodes

The search for materials for thermal computation (thermal diodes, thermal transistors, and thermal logic gates) to develop the so-called phononics (an analogous of electronics, but controlling phonon flow instead of electron flow) has been a stimulus for materials allowing for heat control [115,116].

**Figure 5:**
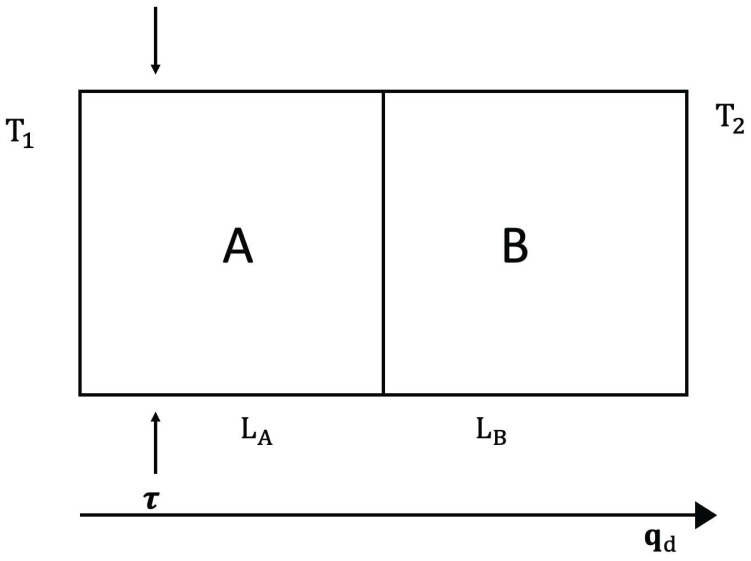
Stress-induced heat rectification. Direct situation.

**Figure 6:**
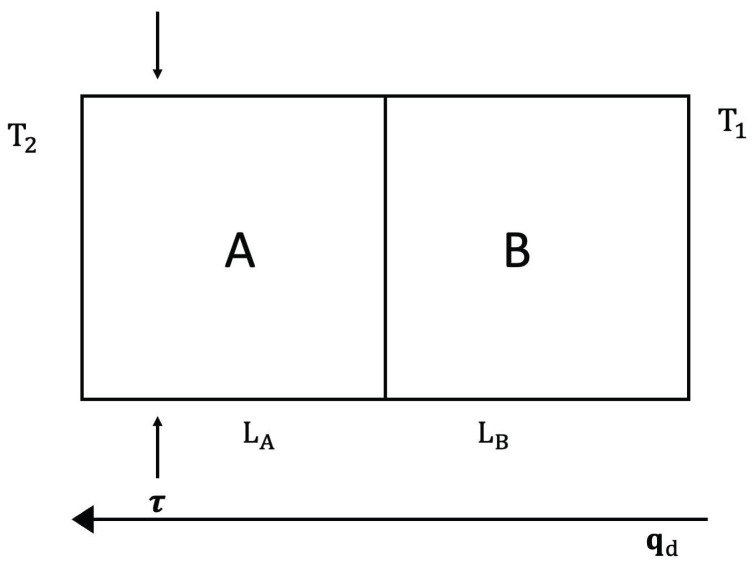
Stress-induced heat rectification. Reverse situation.

First, we consider thermal diodes, which are heat-rectifying devices, in systems with defects, and in systems submitted to a differential external stress along them. Heat rectification consists of an asymmetry of heat transport across a system (see Figure 5 and Figure 6). Namely, application of a temperature gradient in one direction yields for the heat flux a different value than that achieved when the same temperature gradient is applied in the opposite direction. This is analogous to the behavior of rectifying diodes in electronics, though less performant and more difficult to achieve. For this, it is necessary that the thermal conductivity does not only depend on temperature, but also on some other parameters [45].

The rectification coefficient *R* is defined as $R\equiv \frac{q_{d}}{q_{r}}$, for the same couple of values of the temperatures at the boundaries (namely $T_{1}$ and $T_{2}$), but applied in opposite ways, with $q_{d}$ and $q_{r}$ being the values of the heat flux in one direction (direct direction $q_{d}$) and in the opposite direction (reverse direction $q_{r}$). Alternatively, another rectification coefficient may be $R^{\prime }\equiv {\left(T_{1}-T_{2}\right)}_{d}/{\left(T_{1}-T_{2}\right)}_{r},$ for a same value of the heat flux imposed on both directions. If there is symmetry of the heat flux between both directions, one has R=1 (and also $R^{\prime }=1$) and there is no rectification.

We consider a device composed of two materials A and B in contact, submitted to different external stresses or having different defect concentrations.

*Stress-Induced Heat Rectification*

Expression (4) (applied to each material A and B, but with different coefficients and different exponents), allows to control heat flux by changing τ or *c* (see Figure 5 and Figure 6). First, we assume we change τ. Imposing a heat flux *q* in the direct situation (let us say $T_{1}\left(>T_{2}\right)$ on the left surface and $T_{2}$ on the right surface) one has:

(10) $$q=\frac{\lambda_{A}}{L_{A}}\left[1-\beta_{2}\frac{T_{1}+T_{C}}{2}\left(\tau -\tau_{0}\right)\right]\left[T_{1}-T_{C}\right]=\frac{\lambda_{B}}{L_{B}}\left[T_{C}-T_{2}\right],$$

where $T_{C}$ is the temperature at the interface between *A* and *B*. Imposing a heat flux *q* in the reverse situation (where $T_{1}$ is applied to the right surface and $T_{2}$ to the left one) one has, instead:

(11) $$q=\frac{\lambda_{B}}{L_{B}}\left(T_{1}-T_{C}^{\prime }\right)=\frac{\lambda_{A}}{L_{A}}\left[1-\beta_{2}\frac{T_{C}^{\prime }+T_{2}^{\prime }}{2}\left(\tau -\tau_{0}\right)\right]\left[T_{C}^{\prime }-T_{2}^{\prime }\right],$$

where $T_{C}^{\prime }$ and $T_{2}^{\prime }$ are the temperatures at the interface and at the cold border, respectively. One must obtain $T_{C}$ and $T_{2}$ (respectively, $T_{C}^{\prime }$ and $T_{2}^{\prime }$) for given values of $T_{1}$ and *q*. These results are, in the direct case:

(12) $$T_{1}-T_{2}=q\left[\frac{L_{A}}{\lambda_{A}}+\frac{L_{B}}{\lambda_{B}}\right]+\frac{\beta_{2}}{2}\left(\tau -\tau_{0}\right)\left(T_{1}^{2}-T_{1}T_{0}\right)$$

and in the reverse case:

(13) $$T_{1}-T_{2}^{\prime }=q\left[\frac{L_{A}}{\lambda_{A}}+\frac{L_{B}}{\lambda_{B}}\right]+\frac{\beta_{2}}{2}\left(\tau -\tau_{0}\right)\left[T_{1}^{2}-2q\frac{L_{B}}{\lambda_{B}}T_{1}+q^{2}\frac{L_{B}^{2}}{\lambda_{B}^{2}}\right].$$

These results are valid up to the first order in $\beta_{2}$ (the general expressions are cumbersome and do not add new conceptual information).

From (12) and (13), it is seen that rectification arises, namely $R^{\prime }\equiv \left(T_{1}-T_{2}\right)/\left(T_{1}-T_{2}^{\prime }\right)$ is different from 1. Alternatively, one may impose $T_{2}^{\prime }=T_{2}$ and obtain from (13) the value of $q^{\prime }$ (reverse heat flux) corresponding to $T_{1}-T_{2}.$ It is seen that $q^{\prime }\neq q$, and therefore, $R\equiv \frac{q}{q^{\prime }}\neq 1.$ The model we have presented here is a very simplified one, to illustrate only the conceptual bases. The reader will find some more realistic and detailed analyses in [117,118,119,120,121]. Though here, we have assumed two regions A and B with different concentrations of defects and separated by an interface, thermal rectification may also be achieved in systems with a graded distribution of defects, without a discontinuous interface [21,118,119,120,121,122,123,124].

*Defect-induced heat rectification*

Analogous results arise when the effects of defect concentration are considered, instead of those of the applied stress (see also [45]). In this case, assuming $c_{A}\neq 0$ and $c_{B}=0$, one has, for the direct case:

(14) $$q=\frac{\lambda_{A}}{L_{A}}\left[1-\beta_{1}\frac{T_{1}+T_{C}}{2}c_{A}\right]\left[T_{1}-T_{C}\right]=\frac{\lambda_{B}}{L_{B}}\left[T_{C}-T_{2}\right],$$

and for the reverse case:

(15) $$q=\frac{\lambda_{B}}{L_{B}}\left(T_{1}-T_{C}^{\prime }\right)=\frac{\lambda_{A}}{L_{A}}\left[1-\beta_{1}\frac{T_{C}^{\prime }+T_{2}^{\prime }}{2}c_{A}\right]\left[T_{C}^{\prime }-T_{2}\right].$$

The corresponding results for $T_{1}-T_{2}$ (direct) and $T-T_{2}^{\prime }$ (reverse) are, respectively:

(16) $$T_{1}-T_{2}=q\left[\frac{L_{A}}{\lambda_{A}}+\frac{L_{B}}{\lambda_{B}}\right]+\frac{\beta_{1}c_{A}}{2}T_{1}^{2},$$

and

(17) $$T_{1}-T_{2}^{\prime }=q\left[\frac{L_{A}}{\lambda_{A}}+\frac{L_{B}}{\lambda_{B}}\right]+\frac{\beta_{1}c_{A}}{2}\left[T_{1}^{2}-q^{2}\frac{L_{B}^{2}}{\lambda_{B}^{2}}\right].$$

Again, it is seen that for a same *q*, the direct and reverse temperature differences $T_{1}-T_{2}$, $T_{1}-T_{2}^{\prime }$ are different, and that for a same $T_{1}-T_{2},$ the values *q* for the direct and reverse heat fluxes are different.

To these illustrative but elementary calculations, one should add the contribution of the thermal resistance of the interface, which would imply that instead of the temperature $T_{C}$ at the interface, one should consider two temperatures $T_{CA}$ and $T_{CB}$, with $T_{CA}-T_{CB}=R_{AB}q$, $R_{AB}$ being the thermal resistance of the interface, as defined in (7).

For more realistic situations and more detailed analyses beyond this simplified conceptual introduction presented in this subsection, see for instance [90,115,122,123,124,125,126,127,128,129].

### 3.2. Negative Differential Thermal Conductivity, Thermal Transistors

**Figure 7:**
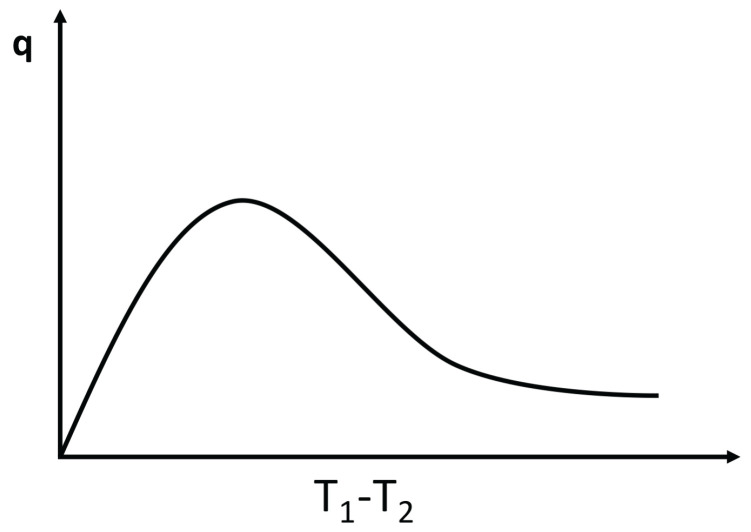
A sketch of *q* as a function of $T_{1}-T_{2}$, showing a nonmonotonic behavior. The zone to the right of the maximum exhibits a negative differential thermal conductivity.

Expression (4) may lead to negative differential heat conductivity in some range of temperatures (see Figure 7). This means that when the temperature difference was applied to the system, $T_{1}-T_{2}$, *q* does not always increase, but it reaches a maximum for a critical $T_{1}-T_{2}$, beyond which it diminishes (this is referred to as negative differential thermal conductivity, because $\frac{dq}{d(T_{1}-T_{2})}$ becomes negative at that regime). Negative differential thermal conductivity is needed to have thermal transistors (see also [45]). Thermal transistors are a very active topic nowadays, with a variety of physical realizations [130,131,132,133,134,135,136,137].

Assume, as in (4), $\lambda \left(T,c\right)=\lambda_{0}\left[1-\beta T^{a}c^{b}\right].$ We impose $T_{2}$ at one boundary and increase $T_{1}\left(>T_{2}\right)$ at the other boundary (or vice versa). The heat flux is $q=\lambda_{0}\left[1-\beta c^{b}\frac{T_{1}^{a}+T_{2}^{a}}{2}\right]$$\left[T_{1}-T_{2}\right].$

The maximum of *q* with respect to $T_{1}-T_{2}$ *is* given by $\frac{dq}{dT_{1}}=0$. Since $T_{2}$ is kept fixed in this illustration, the value of $T_{1}$ corresponding to the maximum of *q* is as follows:

(18) $$\left(1+a\right)T_{1}^{a}-aT_{2}T_{1}^{a-1}=2T_{2}^{a}-\left(2/\beta c^{b}\right).$$

where the value of the maximum depends on the defect concentration *c*, the exponent *a*, the coefficient β, and $T_{2}$. For a=0, there is no solution of (18) and *q* as a function of $T_{1}-T_{2}$ is monotonously increasing.

**Figure 8:**
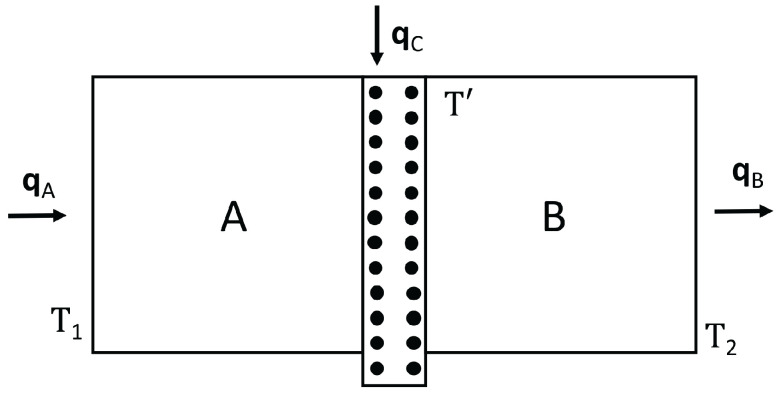
Thermal transistor. The configuration sketched in the figure will work as a thermal transistor if the variations of the outgoing flux, $q_{B}$, are higher than those of the control flux $q_{C}$, i.e., if $\frac{\partial q_{B}}{\partial q_{C}}>1$.

In a thermal transistor (see Figure 8), heat flow $q_{C}$ is introduced in a region of the system, in which a heat flow $q_{A}$ is entering through the hot border at $T_{1}$.

The heat flow $q_{B}$ leaving the system through the cold border at $T_{2}$ is $q_{B}=q_{A}+q_{C}$. In order that variations of $q_{C}$ are amplified, it is needed that $\frac{\partial q_{B}}{\partial q_{C}}>1$. Since $\frac{\partial q_{B}}{\partial q_{C}}=1+\frac{\partial q_{A}}{\partial q_{C}}$, this means that $\frac{\partial q_{A}}{\partial q_{C}}$ must be positive. The relation between heat flow (heat per unit time) and heat flux (heat per unit time and unit area) is the surface through which the heat flow is supplied or extracted, but we do not explicitly write this factor, in order to have simpler expressions.

In the steady state in the device we are considering, we have:

(19) $$q_{C}=q_{B}-q_{A}=\frac{\lambda_{B}}{L_{B}}\left[T^{\prime }-T_{2}\right]+\frac{\lambda_{A}}{L_{A}}\left[T^{\prime }-T_{1}\right]\left[1-\beta \frac{T_{1}^{a}+T^{\prime a}}{2}c^{b}\right].$$

This yields the temperature at the interface $T^{\prime }$ as a function of $q_{C}$, $T_{1}$ and $T_{2}$. Since:

(20) $$q_{A}=\frac{\lambda_{A}}{L_{A}}\left[1-\beta \frac{T_{1}^{a}+T^{\prime a}}{2}c^{b}\right]\left[T_{1}-T^{\prime }\right],$$

one may combine (19) and (20) to obtain $\frac{\partial q_{A}}{\partial q_{C}}=\left(\frac{\partial q_{A}}{\partial T^{\prime }}\right)\left(\frac{\partial T^{\prime }}{\partial q_{C}}\right).$ From here, one has:

(21) $$\frac{\partial q_{A}}{\partial q_{C}}=\frac{\frac{-\lambda_{A}}{L_{A}}+\frac{\beta c^{b}}{2}\frac{\lambda_{A}}{L_{A}}\left(a+1\right)T^{\prime a}}{\frac{\lambda_{B}}{L_{B}}+\frac{\lambda_{A}}{L_{A}}+\frac{\beta c^{b}}{2}\frac{\lambda_{A}}{L_{A}}\left[aT^{\prime a-1}\left(T_{1}-T^{\prime }\right)-T_{1}^{a}-T^{\prime a}\right]}.$$

Note that if β=0, this expression is negative and there is no amplification of the $q_{C}$ variations. Expression (21) may be positive or negative depending on the values of $T^{\prime },\beta ,a,c^{b}$, and so on, so that a detailed analysis must be made in the several cases. For instance, for a=1, expression (21) will be higher than 1 (amplification) for $\beta c^{b}T^{\prime }>1+\frac{1}{2}\left(\lambda_{B}L_{A}/\lambda_{A}L_{B}\right)$. In synthesis, the concrete form of λ(T,c) is a crucial feature regarding the possibility of having or not a thermal transistor with this kind of material. Then, controlling the defect concentration *c* will be important to have a thermal transistor. For this, it is convenient that the region A has a domain of $T_{1}-T^{\prime }$ with negative differential conductivity. Indeed, when increasing $q_{C}$, $T^{\prime }$ will increase, and, since $T_{1}$ is constant, this will reduce $T_{1}-T^{\prime }$. In the usual cases, this reduces $q_{A}$. However, in a region in which differential thermal conductivity is negative, reducing $T_{1}-T^{\prime }$ will increase $q_{A}$, thus leading to $\frac{\partial q_{A}}{\partial q_{C}}>0$.

### 3.3. Efficiency of Thermoelectric Energy Conversion

**Figure 9:**
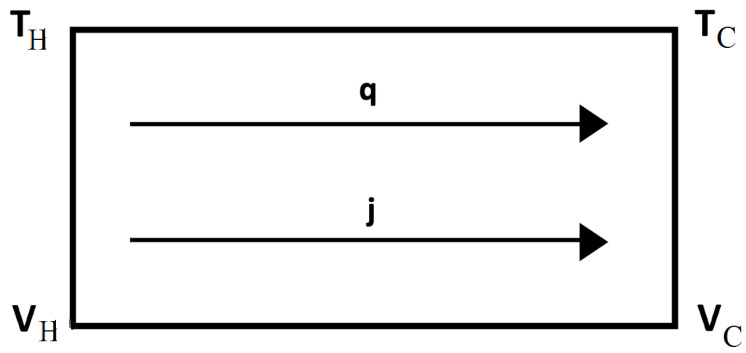
Thermoelectric energy conversion: a fraction of the heat flowing from $T_{H}$ to $T_{C}$ is converted into electric power.

Thermoelectric energy conversion is the partial transformation of a heat flow along a system between temperatures $T_{H}$ (hot) and $T_{C}$ (cold) into an electric flow between electrical potentials $V_{H}$ and $V_{C}$, thus leading to a (partial) conversion of internal energy into electric energy [138] (see Figure 9). The current efficiency of such transformation is still rather low. However, since the amounts of heat lost in industrial and domestic processes are very high, the possibility of using a part of such heat to produce electric energy in small and adaptable devices (for instance, in the escape tubes of cars) would be extremely welcome from economic and environmental points of view. Research on materials and devices optimizing the efficiency of thermoelectric energy conversion is a very active topic, in view of its practical interest [139,140,141,142,143,144,145,146,147,148,149].

The efficiency of thermoelectric energy conversion is the ratio between the electrical power extracted from the system, $i(V_{H}-V_{C})$, and the heat flow (thermal power) entering the system per unit time, namely:

(22) $$\eta \equiv \frac{i(V_{H}-V_{C})}{q}.$$

In (22), *i* is the electric current through the system and $V_{H}$ and $V_{C}$ are the values of the electric potential at left and right sides, which are, respectively, at temperatures $T_{H}$ and $T_{C}$. The equations linking q and j (the intensity i divided by the transversal area of the system) to ∇T and ∇V are as follows:

(23) j=σ∇V−σξ∇T,

(24) $$q=P_{\nabla }T\nabla V-\lambda \nabla T,$$

where ξ and $P_{\nabla }$ are the Seebeck and Peltier coefficients and σ the electric conductivity. In particular, the Seebeck coefficient (describing the Seebeck effect, namely, the appearance of an electric potential gradient in a system when a temperature gradient is imposed on it) relates the value of $V_{H}-V_{C}$ to the value of $T_{H}-T_{C}$ when the electric flux j is zero. The Peltier coefficient $P_{\nabla }$ is related to ξ through $P_{\nabla }T=\xi$, which is a particular case of Onsager relations.

For readers not familiar with nonequilibrium thermodynamics, it may be useful to recall that the Onsager reciprocity relations (shown by Onsager in [150,151]) state that the phenomenological coefficients relating thermodynamic fluxes to thermodynamic forces are symmetrical. This means that if $J_{i}$ are the fluxes and $X_{j}$ are the conjugate thermodynamic forces, in such a way that the entropy production is a bilinear expression $J_{i}X_{i}$ (with summation over repeated subindices), and the fluxes are written in terms of the forces as $J_{i}=L_{ij}X_{j}$, with summation with respect to subindex *j* and with $L_{ij}$ phenomenological transport coefficients, then $L_{ij}=L_{ji}$ [55,71,72,73,152,153].

The Seebeck coefficient may be positive or negative, depending on whether negative charges (electrons) or positive ones (holes) are dominant in the electric transport (i.e., in *n* or *p* doped semiconductors, respectively).

By applying (23) and (24) to a rod of thermoelectric material between heat reservoirs at temperatures $T_{H}$ and $T_{C},$ it is found that the maximum value of (22) is as follows:

(25) $$\eta_{max}=\frac{T_{H}-T_{C}}{T_{H}}\frac{{\left(1+ZT_{av}\right)}^{1/2}-1}{{\left(1+ZT_{av}\right)}^{1/2}+\left(T_{C}/T_{H}\right)},$$

where $\frac{T_{H}-T_{C}}{T_{H}}$ is the Carnot efficiency between $T_{H}$ and $T_{C}$, $T_{av}$ is the average temperature of the rod between $T_{H}$ and $T_{C}$, and ZT is the dimensionless combination $\;ZT=\frac{\xi^{2}\sigma }{\lambda }T,$ which is the so-called figure of merit. Note that λ is $\lambda =\lambda_{p}+\lambda_{e},$, where $\lambda_{p}$ and $\lambda_{e}$ are the phonon and electron contributions to the thermal conductivity, respectively. Therefore, reducing $\lambda_{p}$ (as in superlattices, for instance, where the interfaces become barriers to the phonons, or in nanowires, where the collisions of phonons with the walls increase the thermal resistance) increases ZT. An increase in ZT enhances the value of $\eta_{max}$ in (25).

Trying to increase the currently available values of ZT (note that *Z* depends on *T*) is one of the practically relevant aims of material engineering in this field. Some particularly interesting systems are $Bi_{2}Te_{3}$ and $Bi_{2}Se_{3}$, with ZT between 0.8 and 1, and only mildly dependent on *T*. In contrast, superlattices of thin alternating layers of these materials may achieve values of ZT from 1.5 to 2.0 at room temperature. PbTe doped with Thallium reaches ZT=1.5 at 770 K, whereas doped with Selenium, $\left(PbTe_{1-x}Se_{x}\right)$ has been reported to reach ZT=2.2, the maximum value achieved up to now. PbTe and PbTeSe superlattices obtain ZT=1.5 at room temperature. Thus, it is seen that both defects (in this case, doping atoms) and nanostructures (thin-layer superlattices, quantum-dot superlattices, nanostructured alloys) do indeed increase ZT, mainly because of a reduction of the phonon contribution to the thermal conductivity without substantially reducing the electronic conductivity, because the mean free path of electrons is much smaller than that of phonons.

However, increasing ZT is not enough to ensure an optimal global efficiency for the thermoelectric converter along the mentioned converting rod, whose temperature changes along the rod. To achieve a global maximum, it must be taken into account that *Z* as a function of *T*, Z(T), has a relatively narrow maximum as a function of temperature, because usually, σ/λ increases with *T* whereas ξ decreases with T. Thus, the maximal value of the efficiency (25) will be reached only at a narrow region if the system consists of a single material. Instead, if it is made of a succession of two (or more) suitable materials A and B, each of them with their own maximum of ZT at different temperatures, the local maximum of η may be reached at two (or more) points, thus, contributing to an increase of the global maximum along the whole rod.

*A Simplified Illustration*

To illustrate in a simplified way this topic, let us assume that all coefficients in (23) and (24) are reduced by the concentration of defects as follows:

(26) $$\lambda =\frac{\lambda_{0}}{1+\beta T^{a}c^{b}};\;\sigma =\frac{\sigma_{0}}{1+\beta_{e}T^{k}c^{l}};\;\;\xi =\frac{\xi_{0}}{1+\beta_{s}T^{m}c^{n}},$$

namely, the several transport coefficients decrease with increasing *c*. However, if the reduction of λ is higher than the reduction of $\xi^{2}\sigma$, the value of ZT will increase after reaching a sufficiently high value of *c*.

Then, up to the first order in the c-dependent term, one has for ZT:

(27) $$ZT=\frac{\xi_{0}^{2}\sigma_{0}}{\lambda_{0}}T\left[1+\beta T^{a}c^{b}-\beta_{e}T^{k}c^{l}-2\beta_{s}T^{m}c^{n}\right].$$

The maximum of ZT will correspond to $\frac{d(Z(T)T}{dT}=0,$ leading to:

(28) $$1+\left(1+a\right)\beta T^{a}c^{b}-\left(1+k\right)\beta_{e}T^{k}c^{l}-2\left(1+m\right)\beta_{s}T^{m}c^{n}=0.$$

This indicates that $T_{max}$, i.e., the temperature at which the maximum of ZT is achieved, depends on the concentration *c* of defects. Note that, since the thermal conductivity in (26) depends on *c*, the temperature profile will also depend on the concentration profile.

In summary, one should try to maximize ZT as much as possible at each point. One way of achieving this would be changing the concentration of defects (or alternatively, changing the material) along the system, in such a way that the temperature T(z) along the longitudinal axis of the rod fits the local optimum value $T_{max}$ (given by the maximum of ZT) in a convenient way along the system. This is what is known as functionally graded materials, namely, a succession of materials fitting some particular combination of material properties leading to the optimization of some function.

*Thermoelectric Refrigeration*

**Figure 10:**
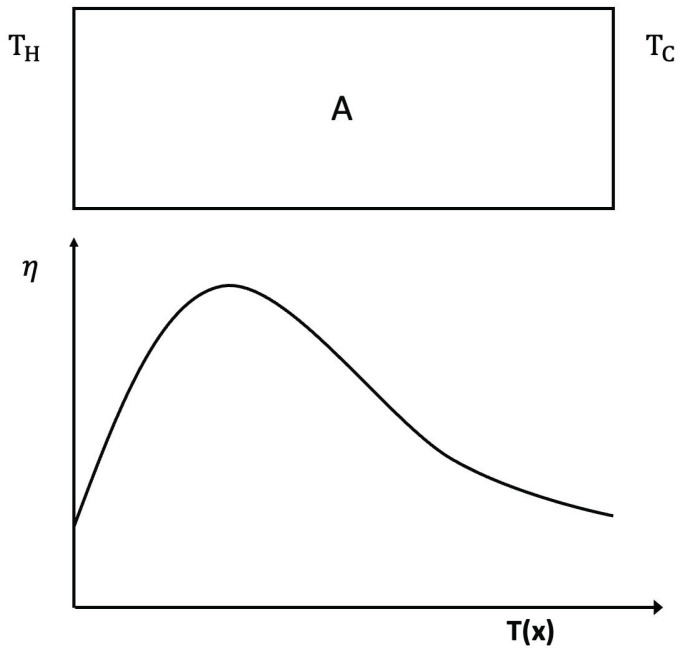
Single-material thermoelectric energy converter: local efficiency versus position. Since ZT has a narrow maximum at a given temperature, and since temperature depends on the position, the maximumm efficiency will only be achieved in a narrow region.

**Figure 11:**
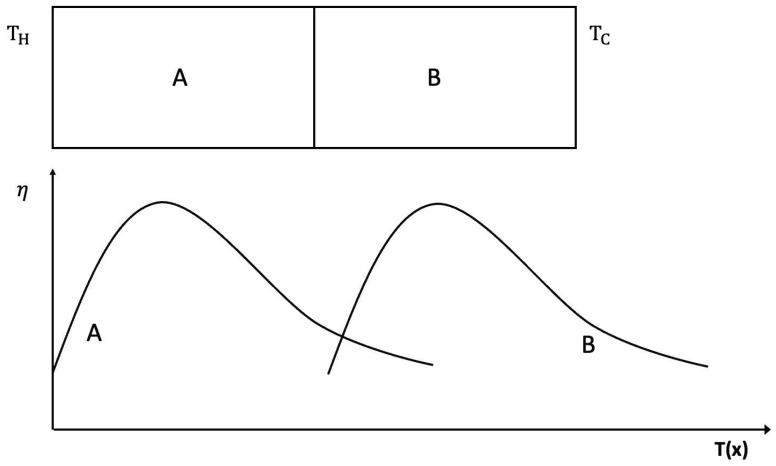
Two-material thermoelectric energy converter: local efficiency versus position. For each system (A and B), ZT reaches the maximum at a given temperature dependent on the material. If the system were only composed of material A or of material B, the maximum thermoelectric efficiency would be achieved only in a single narrow region.

A situation complementary to thermoelectric energy conversion is thermoelectric refrigeration, based on the Peltier effect instead of the Seebeck effect (see Figure 10 and Figure 11). The Peltier effect consists of a heat absorption or emission through an interface between two different materials when an electric current flows between them. If the current flows in one direction, heat is absorbed at the interface, and if the current is reversed, heat will be emitted at the interface. This effect may be used to heat or to cool a system. Though the effect through a single interface is small, one may use a series of alternating materials ABABAB… in such a way that current from A to B absorbs heat and current from B to A emits heat. Putting the interfaces AB in contact with the system to be cooled and the interfaces BA in contact with the environment, one may extract heat from the system and bring it to the environment, or vice versa.

This procedure has the advantages of not requiring mobile parts (and thus, being less vulnerable to mechanical problems) and of being miniaturizable. These features make it of much interest in miniaturized technologies, as for instance to remove heat from miniaturized chips of computers, where the reduction of the effective thermal conductivity at the nanoscale makes it very difficult to remove heat through classical strategies based on heat conduction.

*Effects of Applied Stresses to Enlarge the Optimal Behavior*

When designing graded materials to enhance the global efficiency of an elongated thermoelectric energy converter or thermoelectric refrigerator, one takes into consideration the variation of the transport coefficients with *T* and *c*. Taking into consideration the effects of stress could also be helpful to tune the values of such coefficients, if the temperatures $T_{H}$ and $T_{C}$ at the boundaries are modified. The application of lateral stresses has the advantage of being applicable without changing the material, and of being easily modified according to the changing boundary conditions. However, the optimization of the efficiency of the rod by means of a suitable graduation of composition, depends very much on the temperature profile. The possibility of regulating the applied lateral stress along the system would allow to tune the suitable temperature profile. To have a concrete illustration, assume a converter composed of two elements *A* and *B* in series (as in Figure 11).

The total efficiency of the system is:

$$\eta_{total}=\eta_{A}+\eta_{B}\left(1-\eta_{A}\right),$$

where $\eta_{A}$ and $\eta_{B}$ are the respective efficiencies of the elements *A* and *B*.

If $T_{C}$ (the temperature at the cold boundary) is changed, there will be a modification in $\eta_{total}$, i.e.,

$$\frac{d\eta_{total}}{dT_{C}}=\frac{d\eta_{A}}{dT_{C}}\left(1-\eta_{B}\right)+\frac{d\eta_{B}}{dT_{C}}\left(1-\eta_{A}\right).$$

If $Z_{A}^{T}$ and $Z_{B}^{T}$ depend only on $T,\;c_{A}$ and $T,\;c_{B}$, and if $c_{A}$ and $c_{B}$ have been chosen to optimize the efficiency for given $T_{H}$ and $T_{C}$, respectively, a change in $T_{C}$ will decrease the efficiency of the thermoelectric conversion. If the transport coefficients depend also on the applied stress τ, the practical problem would be to find the extra stresses $\tau_{A}$ and $\tau_{B}$ in order that, given $c_{A}$ and $c_{B}$, the reduction in the total efficiency is minimized.

### 3.4. Thermal Cloak and Thermal Concentration

The functions of thermal metamaterials most actively explored in the last fifteen years have been thermal cloaking (protecting a region of space by shielding it of high heat fluxes or of fast variations of the heat flux), and thermal concentration (focusing heat transport towards a given region). Related to these functionalities, there are also thermal inverters, thermal illusions, and temperature trapping devices. Every one of these functions requires a detailed local tuning of an anisotropic thermal conductivity [25,26,27,28,29,30,31,32,33,34,35,36,37,38,39,40].

**Figure 12:**
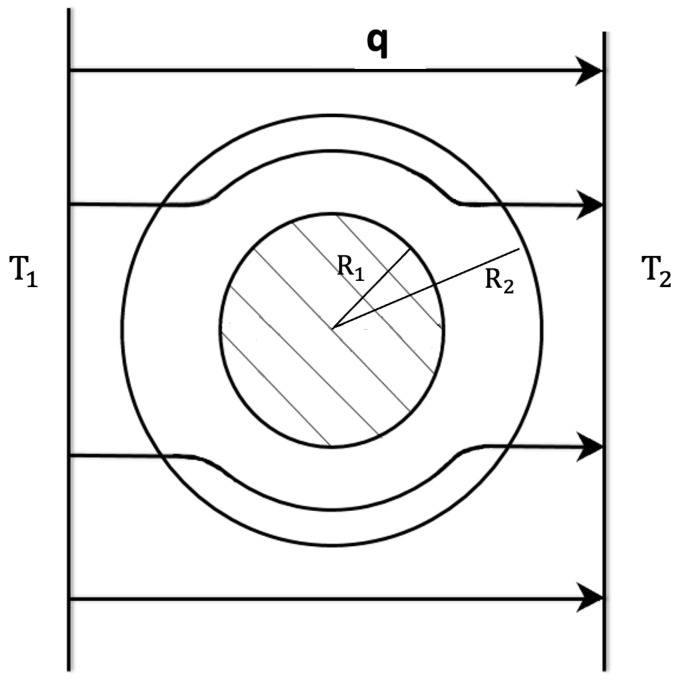
Thermal cloak: A sketch of a cylindrical thermal cloak. When put between two parallel plates at temperatures $T_{1}$, $T_{2}$$\left(<T_{1}\right)$, the heat flows along the cylindrical walls in such a way that it does not penetrate in the inner cavity (thermal protection), and that the heat flux distribution at the plate at $T_{2}$ is the same as if the material between the plates were perfectly homogeneous (thermal invisibility).

A kind of thermal cloak extensively studied has a cylindrical form (see Figure 12). The walls are composed of concentric layers of suitable thermal conductivities. When the cloak is introduced between two parallel plates at different temperatures, it deviates the heat flow along its cylindrical walls, leaving practically unaffected the spatial distribution of the heat flux arriving to the cold wall, and preventing the heat flow from penetrating into the inner cavity. The first effect is usually known as “thermal invisibility”, because an external observer watching only the cold wall would not be able to decide whether or not there is an object between the two plates; the second effect is shielding the system in the inner cavity (see Figure 12) from heat fluxes [28,31].

To achieve a cylindrical thermal cloak of annular form with radius $R_{1}$ to $R_{2}$ ($R_{2}>R_{1}$) in an homogeneous surrounding material with thermal conductivity $\lambda_{0}$, the radial conductivity of the annular region of radius *r* is given by:

(29) $$\lambda_{rr}\left(r\right)=\lambda_{0}{\left(\frac{R_{2}}{R_{2}-R_{1}}\right)}^{2}{\left(\frac{r-R_{1}}{R_{1}}\right)}^{2}<\lambda_{0},$$

whereas the azymuthal component $\lambda_{\vartheta \vartheta }$ must be constant, given by:

(30) $$\lambda_{\vartheta \vartheta }=\lambda_{0}\left(\frac{R_{2}}{R_{2}^{2}-R_{1}^{2}}\right)>\lambda_{0}.$$

This particular distribution is mathematically obtained by transforming the isotherm curves solution of the homogeneous problem $\nabla \cdot \left[\lambda_{0}\left(T\right)\nabla T\right]=0$ into the solutions of $\nabla \cdot \left[\lambda (r,\vartheta ,T)\cdot \nabla T\right]=0,$ with:

(31) $$\lambda \left(r,\vartheta ,T\right)=\left|\left|\begin{array}{cc} \lambda_{rr}\left(r,\vartheta ,T\right) & 0\\ 0 & \lambda_{\vartheta \vartheta }\left(r,\vartheta ,T\right) \end{array}\right|\right|,$$

to be determined in the annular region $R_{1}\leq r\leq R_{2}$, by keeping the thermal conductivity $\lambda_{0}\left(T\right)$ for $r<R_{1}$, and $r>R_{2}$. Once the mathematical form of λ(r,ϑ,T) is specified, the practical problem is building a material which has at every point the required value of λ(r,ϑ,T). In general, these materials do not exist in nature, and they must be designed and manufactured. Since the conductivity must be anisotropic, this implies much more complex requirements than achieving particular local values of scalar thermal conductivity, as in a usual functional graded system. One way of achieving the radial distribution (see (29) and (30)) of the thermal conductivity is by means of a superlattice of concentric annular layers of materials of suitable conductivities (for instance alternative layers of good thermal conductors with layers of bad heat conductors, whose combination in different proportions allows to increase or decrease the conductivity). The suitable radial distribution (29) could also be achieved by an annular graded system with a variation of the composition as a function of the radius; this is more difficult to achieve practically than the multilayered structure.

**Figure 13:**
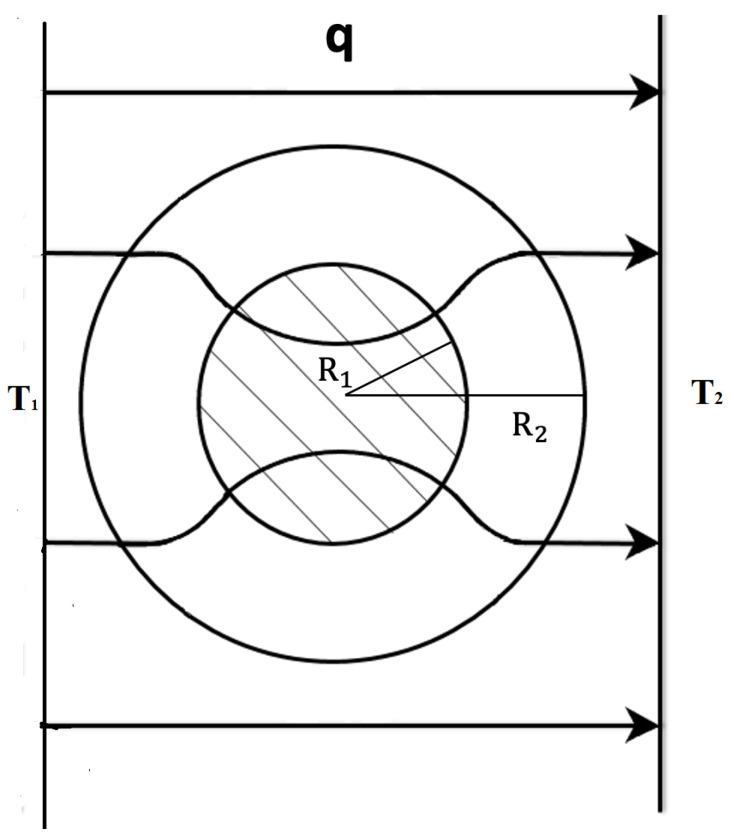
Thermal concentrator. In contrast to the thermal cloak in the previous figure, which implied zero heat flux in the central cavity of the cylinder, the thermal concentrator increases the heat flux in the center. This requires a different spatial distribution of the thermal conductivity than in a thermal cloak.

Another interesting functionality is the thermal concentration (see Figure 13), namely, a concentration of heat flow in the inner region, instead of shielding it from the external heat flow, as in the thermal cloak. To achieve this, the relation between the radial and azymuthal components of λ, namely $\lambda_{rr}$ and $\lambda_{\vartheta \vartheta }$, must satisfy:

(32) $$\frac{\lambda_{rr}}{\lambda_{0}}=\frac{\lambda_{0}}{\lambda_{\vartheta \vartheta }}.$$

There are two main ways of achieving this: one is by means of alternating radial sectors of different materials, while the other one is by inserting small ellipsoidal inclusions of a material of suitable conductivity, with the values of the axes of the ellipsoids being carefully calculated. In fact, ongoing research focuses on bifunctional devices, such that they may act as thermal cloaks or as thermal concentrators, by acting on the system [25,26]. The control of thermal conductivity and of electrical conductivity also allows independent manipulation of heat and electrical current bifunctional metamaterials. The material and geometrical features must be combined to achieve convenient relations of anisotropy [25].

**Figure 14:**
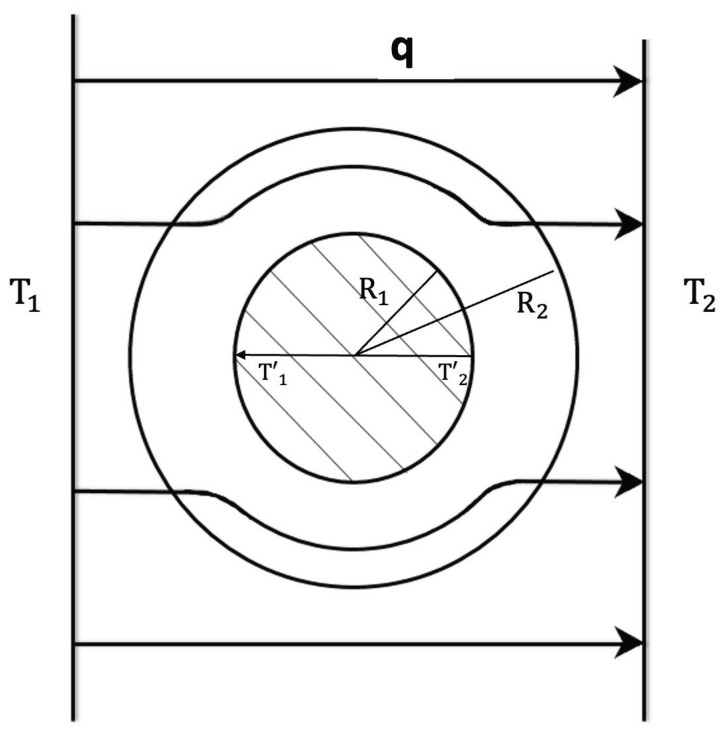
Thermal inverter. The device in the figure ensures that the heat flux in the central cavity has a direction opposite to the heat flow outside, as if the thermal conductivity were negative in this region. In reality, this is not so, but the temperature is distributed in such a way that inside the region, $T_{1}^{\prime }<T_{2}^{\prime }$, in contrast to the external one, where $T_{1}>T_{2}$.

A third configuration of interest is the so-called thermal inverter, which achieves that inside the thermal cavity of the device (see Figure 14), heat flows in an opposite direction to that of the external flow. This is achieved on the side 1 of the annular region of a small conductivity, where the internal temperatures $T_{1}^{\prime }$ and $T_{2}^{\prime }$ on the central axis are such that $T_{1}^{\prime }<T_{2}^{\prime }$, despite the external temperatures being $T_{1}>T_{2}$.

### 3.5. Oscillations and Wave Propagation

In Section 3.1, Section 3.2, Section 3.3 and Section 3.4, we have considered some steady-state phenomena. Time-varying phenomena are also of much interest. For instance, an analysis of wave propagation of perturbations in an equilibrium reference system provides information on many of the coefficients appearing in the constitutive equations. Further information may be obtained on the coefficients of nonlinear terms by studying wave propagation in nonequilibrium steady states submitted to a constant heat flux.

In particular, heat waves, ultrasound waves, and thermoelastic waves propagation have been much studied [55,154,155,156,157,158,159]. The high-frequency behavior of such waves requires generalizing the classical transport equations in order to incorporate the finite (nonvanishing) value of the relaxation time of the heat flux or of other fluxes. This is the time that it takes for a flux to reach its classical value, after the corresponding thermodynamic force has been suddenly changed. Typical problems are, for instance, obtaining the high-frequency limit of the wave propagation velocity in an equilibrium state or along or against a steady-state heat flux characterizing a nonequilibrium steady state, and obtaining the velocities of thermoelastic waves, when the effect of a nonzero thermal expansion coefficient provides a coupling between thermal effects and elastic effects. These kinds of phenomena require a generalization of Fourier’s law.

Besides high-frequency effects, nonlinear effects for relatively high-amplitude waves are also of much interest, leading to a rich phenomenology about second- and third-harmonic generation, self-focusing of waves, soliton propagation, and so on.

Though in this review we are mainly dealing with solid systems (including fixed or mobile defects), it is also interesting making an analogy with liquids and gases with suspended particles, considered as defects. This is a very rich field in which the particles modify the transport properties of the fluid, and in which the mobility of the particles plays a relevant role. Defects in solids are more static than particles in liquids, since moving inside a solid requires much more energy than in a liquid or a gas. Thus, the coupling between the dynamics of the particles and the heat flux through the influence of particles on specific heat and on thermal conductivity is especially interesting in fluids.

Here, we do not have space enough to deal with such analogies.

## 4. Main Equations

The main new ingredients to be considered in the present paper with respect to the nonequilibrium thermodynamics of heat transport in usual crystal semiconductors are: (a) geometrical structure (parallel interfaces perpendicular to a given axis, anisotropic thermal conductivity), (b) compositional inhomogeneity (graded materials); (c) defects (requiring a scalar variable for point defects, or a tensorial variable in the case of line defects, as for instance, dislocations [160,161]); (d) applied stresses. Whether these quantities must be considered as fixed parameters or as internal variables depends on whether they require an evolution equation for their own, or if they are kept constant.

### 4.1. Selection of Variables

There may be some differences in the treatment of variables in equilibrium thermodynamics and in nonequilibrium thermodynamics. Quantities without an evolution Equation (for instance, a fixed amount of impurities) do not appear as a variable of the entropy in formalisms of nonequilibrium thermodynamics, but they may have much relevance in equilibrium thermodynamics (for instance, in specific heats, thermal expansion coefficients and elastic coefficients, and in the phase diagram and related phase transition temperatures and latent heats) or in transport theory (for instance, through the values of the transport coefficients).

In the systems we are considering in the present paper, the interfaces in a superlattice are usually described by a parameter (the thermal resistance, for instance, depending on the composition of the two materials in mutual contact, or the mechanical resistance, in the case that the materials in contact have different mechanical properties), but they could require an evolution equation of an additional variable in the case they have some particular inertia or whether their internal concentration profile slowly relaxes by diffusion of the atoms of both sides or by chemical reactions. Furthermore, in a graded system, the composition profile may be assumed to not change in time, or it may change with time as a consequence of diffusion and reaction processes, in which case an evolution equation of a suitable variable is needed to describe it.

Point defects and line defects, as the dislocation lines, may also be considered as constant parameters or as internal variables. This is so if they change in time as a consequence of diffusion or of a drift from regions with higher stress to regions with lower stress. Usually, to describe point defects it is sufficient to describe their local density, *c*, namely, a scalar variable. Instead, to describe the local structure of dislocation lines, a tensorial variable is necessary. The trace of this dislocation tensor is the dislocation density $\rho_{D}$ (total length of dislocation lines per unit volume, which is the simplest variable in the description of dislocations). The dislocation lines form a network of infinitesimally thin capillary channels inside an elastic solid. These defects, acquired during a process of fabrication, can self propagate, because of favorable surrounding conditions, provoking a premature fracture of the solid. The dislocation lines disturb the periodicity of the crystal lattice. If in a perfect crystal an extra plan of atoms is inserted, a dislocation line can be created, and the bottom edge of this plane is an edge dislocation. The line along which a lattice plane of the solid undergoes a rotation is called screw dislocation [160]. In [47,76,162,163,164], a dislocation core tensor la Maruszewski [165] and its flux are introduced in the thermodynamical state space of independent variables to describe these defects. Here, see [42], a new dislocation tensor is used, defined by the authors in [41], to describe the geometry and the orientation of these defect lines. Let us note that an analogous kind of tensor is defined to describe the quantized vortices in turbulent superfluid Helium II [166]. Thus, let us consider a representative elementary volume Ω of a structure with dislocations, large enough to provide a representation of all the statistical properties of this volume, where the dislocations resemble a network of infinitesimally thin channels. All the microscopic quantities are given with respect to the $\xi_{i}$ coordinates (*i* = 1, 2, 3), describing the position along a given dislocation line, while the macroscopic quantities are described with respect to the $x_{i}$ coordinates (*i* = 1, 2, 3), with *i* spatial index relates to Descartes coordinates. Thus, the dislocation lines density, $\rho_{D}$, is given by the average length of dislocation lines per unit volume $\rho_{D}=\frac{1}{\Omega }\int dl,$, where dl is the elementary length element along the dislocation lines and the integration is carried out over ξ and over all dislocation lines present in the elementary local volume Ω at x. The dislocation field is described by the microscopic tensor [41]: a(ξ)≡n(ξ)⊗n(ξ), where n(ξ) is the tangent unit vector along the dislocation line at position ξ (an analogous kind of tensor has been used for the description of quantized vortices in turbulent superfluid helium II [166]). We define the macroscopic dislocation tensor a(x) as the local average of a(ξ) in the following way:

(33) $$a_{ij}\left(x\right)=\langle a(\xi )\rangle =\frac{1}{\Omega }\int_{\Omega }n_{i}\left(\xi \right)n_{j}\left(\xi \right)dl,$$

where 〈…〉 represents the average calculated on the ensemble of lines inside the elementary volume Ω. The components $a_{ij}$ model the anisotropy of the dislocation lines, and have unit $m^{-2}$. Note that the tensor (33) is symmetric (see [165] for a different definition of a(ξ)).

The objects of our study are semiconductors [167,168,169,170,171,172,173,174], semiconductor superlattices, graded semiconductors, and thermal metamaterials, with point defects or line defects, which may be static or mobile, neutral or charged, and with applied mechanical stresses. The fields acting inside the system are (see [41,42,47,162,163,164]):

(i) The electromagnetic field, described by the electric field $E_{i}$ and the magnetic induction $B_{i}$; this is of interest when the system is electrically charged or polarized, or in the case one wants to study the interaction between electromagnetic radiation and the material system; $E_{i}$ is the electric field in the comoving frame related to $E_{i}$ and $B_{i}$ as $\;E_{i}=E_{i}+\varepsilon_{ijk}v_{j}B_{k}$, where $\varepsilon_{ijk}$ is the antisymmetric Levi-Civita pseudo-tensor and $v_{i}$ the body point velocity.

(ii) The elastic field, described by the stress tensor $\tau_{ij}$ and the small-strain tensor $\varepsilon_{ij}$, defined by $\varepsilon_{ij}=\left(1/2\right)\left(u_{i,j}+u_{j,i}\right)$, where $u_{i}$ is the displacement vector and a comma in lower indices is for spatial derivation; a plastic field could also be required in the case that the motion of dislocations would be high enough.

(iii) The thermal field, described by the temperature *T* and its gradient.

(iv) The charge-carrier fields, described by the concentrations of electrons *n* and holes *p*, their gradients $n_{,i}$, $\;p_{,i}$, and their currents $j_{i}^{n}$ and $j_{i}^{p}$, respectively; in principle, if we are interested in inhomogeneous systems (as graded systems, for instance) or in nonlocal effects, the spatial gradients of *n* and *p* are also considered.

(v) The defect fields, described by the dislocation density tensor $a_{ij}$ and its gradient $a_{ij,k}$; in the case of point defects, a scalar variable would be sufficient (point density c).

The results we give here referring to dislocation density can be formally applied to point defect density using only the trace of the tensor, which is a scalar.

Besides these fields, one may introduce in the state space as additional independent variables the respective fluxes $j_{i}^{n},j_{i}^{p},q_{i}$, and $V_{ijk}$, in the case that their relaxation properties are taken into account; however, we ignore the relaxation properties of the mechanical field, so that $\tau_{ij}$ is not in the set *C* and the viscoelastic or viscoplastic properties of the medium are excluded. It is assumed, for the sake of simplicity, that the interfaces between the alternating semiconductors are given and remain static, so that they are described by parameters rather than by independent variables. The set of the independent variables is, therefore:

(34) $$C=\left\{E_{i},B_{i},|\varepsilon_{ij},n,p,T,a_{ij},\left|n_{,i},p_{,i},T_{,i},a_{ij,k},\right|j_{i}^{n},j_{i}^{p},q_{i},V_{ijk}\right\}.$$

### 4.2. Governing Equations

To describe the evolution of the system, three groups of laws have to be considered (see [41,42,47,76,162,163,164]). The first group concerns the *balances of mass, momentum, charges, and energy*, describing a wide variety of materials without specifying the concrete material properties.

The *continuity equation* is given by:

(35) $$\dot{\rho }+\rho v_{i,i}=0,$$

where the dot over a quantity indicates the material time derivative $\frac{d}{dt}=\frac{\partial }{\partial t}+v_{i}\frac{\partial }{\partial x_{i}}$, where $\frac{\partial }{\partial t}$ is the partial time derivative), ρ denotes the mass density of the whole body, the elastic semiconductor in this model, since the charge carriers have been neglected compared to ρ, and $v_{i}$ is the barycentric velocity of the whole body. In the following, we assume that ρ is constant.

The *momentum balance* has the following form:

(36) $$\rho {\dot{v}}_{i}=\tau_{ji,j}+\rho ZE_{i}+\varepsilon_{ijk}\left(j_{j}^{n}+j_{j}^{p}\right)B_{k}+f_{i},$$

where in the right-hand side, the respective terms describe the elastic force, electric force, magnetic force, and external body force, and *Z* is the concentration of the total charge, which we will define in (38).

The *energy balance* is as follows:

(37) $$\rho \dot{U}=-q_{i,i}+\tau_{ji}\frac{d\varepsilon_{ij}}{dt}+\left(j_{j}^{n}+j_{j}^{p}\right)E_{j}+\rho r,$$

where U is the internal energy density and the right-hand terms correspond, respectively, to the heat exchange, the mechanical work, the electric work, and the heat source.

We define the concentration of the total charge *Z* and the density of the total electric current $j^{Z}$ as follows:

(38) $$Z=n+p,\;j_{i}^{Z}=\rho Zv_{i}+j_{i}^{n}+j_{i}^{p},\;\mathrm{with}\;j_{i}^{n}=\rho n\left(v_{i}^{n}-v_{i}\right),\;j_{i}^{p}=\rho p\left(v_{i}^{p}-v_{i}\right),$$

where ρ denotes the mass density of the whole body, n(n<0) is the concentration of the negative electric charge density (coming from the density of the free electrons given by doping the semiconductor by pentavalent impurities and the density of the intrinsic semiconductor base free electrons), p(p>0) is the concentration of the total positive electric charge (coming from the concentration of the holes produced by doping the semiconductor by trivalent impurities and the concentration of the intrinsic semiconductor base holes), $j^{Z}$ is the density of the total electric current, $\;v_{i}^{n}\;$ and $\;v_{i}^{p}\;$ are the velocities of electric charges *n* and *p*, and $\;j_{i}^{n}\;$ and $\;j_{i}^{p}\;$ are their currents (i.e., the electric currents due to the relative motion of the electric charges respect to the barycentric motion of the body). The sum of these last currents gives the conduction electric current, whereas $\rho Zv_{i}$ is the electric current due to convection.

The *charge conservation laws* are as follows:

(39) $$\rho \dot{n}+j_{i,i}^{n}=g^{n},\;\rho \dot{p}+j_{i,i}^{p}=g^{p},\;g^{n}+g^{p}=0,$$

where $g^{n}$ and $g^{p}$ are source terms, and Equation (39)_3_ describes the recombination of electrons and holes. In this description, we have not taken into consideration the fixed ionized charges coming from impurities. The terms $g^{n}$ and $g^{p}$ may also be related to photon production of electrons and holes (essential in photovoltaic cells) as well as a recombination of electrons and holes emitting electromagnetic radiation (as in LED devices or semiconductor lasers). The control of these quantities is of upmost technological interest, but we will focus on heat and electric transport. We have:

(40) $$\rho \dot{Z}+j_{i,i}^{Z}=0.$$

The second group of laws concerns *Maxwell’s equations* in Galilean approximation, describing the electromagnetic field and having the following form:

(41) $$\varepsilon_{ijk}E_{k,j}+\frac{\partial B_{i}}{\partial t}=0,\;D_{i,i}-\rho Z=0,$$

(42) $$\varepsilon_{ijk}H_{k,j}-j_{i}^{Z}-\frac{\partial D_{i}}{\partial t}=0,\;B_{i,i}=0,$$

where D and H are the electric displacement and the magnetic field per unit volume, respectively. The time variation of E and B would be of central interest for the description of radiative phenomena. Otherwise, E and B may be taken as externally controlled parameters.

Here, we assume that the magnetic and dielectric properties of the semiconductor may be disregarded, so that the magnetization and the polarization of the body are null, and then:

(43) $$\;H_{i}=\frac{1}{\mu_{0}}B_{i},\;E_{i}=\frac{1}{\varepsilon_{0}}D_{i},$$

where $\varepsilon_{0}$ and $\mu_{0}$ are the permittivity and permeability of vacuum, respectively. In [76], a model for a polarized semiconductor was formulated.

The last group of laws concerns the transport equations, which in our case are expressed as *the rate equations for defect evolution, defect flux, charge flux, and heat flux*. In contrast to the previous two groups of equations, which are very general, this third group of equations directly depends on the materials being considered, and they define their particular behaviors. Nonequilibrium thermodynamics focuses on these equations, trying to obtain some information on the general kinds of behaviors allowed by the second law of thermodynamics. In abstract general terms, to be specified below, these laws are chosen in the following form:

(44) $${\dot{j}}_{i}^{n}=J_{i}^{n}\left(C\right),\;\;\;\;\;{\dot{j}}_{i}^{p}=J_{i}^{p}\left(C\right),\;\;\;\;\;{\dot{q}}_{i}=Q_{i}\left(C\right),\;\;\;\;\;{\dot{a}}_{ij}=-V_{ijk,k}+A_{ij}\left(C\right),\;\;\;\;\;{\dot{V}}_{ijk}=V_{ijk}\left(C\right),$$

where *C* refers to the whole set of variables given in (34) and $J_{i}^{n}\left(C\right)$, $J_{i}^{p}\left(C\right)$, $Q_{i}\left(C\right)$, $A_{ij}\left(C\right)$, and $V_{ijk}\left(C\right)$ are functions which specify the transport properties of the system. Here, we assume that $V_{ijk}$ is not an independent variable, but that it is proportional to the gradient of $a_{ij}$. In the rate equations for $j^{n}$, $j^{p}$, and q, higher-order flux terms could also be incorporated, which may be especially relevant in nanosystems, but we avoid this complication.

### 4.3. Second-Law Restrictions

All the admissible solutions of the evolution equations are restricted by the second law of thermodynamics, expressed through the *entropy inequality:*

(45) $$\rho \dot{S}+\Phi_{k,k}-\frac{\rho r}{T}\geq 0,$$

where *S* is the entropy per unit mass and $\Phi_{k}$ the entropy flux. Indeed, the law (45), with its amendment: “Except in equilibrium, there are no reversible process directions in the state space”, states that “all local solutions of the balance equations have to satify the dissipation inequality (45)” [70,71,74,175,176,177].

When integrated over an insulated system (namely, when the entropy flux at the surface is null), Equation (45) implies that the total entropy of the system may only increase or remain constant, but it cannot decrease as a function of time. In fact, this statement is stronger than Clausius’ classical statement of the second law, which states only that the entropy of the final equilibrium state must be higher or equal than the entropy of the initial equilibrium state [178,179,180,181,182,183,184,185,186,187,188,189,190,191]. The entropy *S* is in principle a function of the whole set *C* (34) of independent variables. This *S* is more general than the equilibrium entropy which depends only on conserved variables ($U,\varepsilon_{ij},n,p,a_{ij}$), being also a function of nonequilibrium variables. If the entropy is differentiable, we have:

(46) $$dS=\sum_{i}\frac{\partial S}{\partial \alpha_{i}}d\alpha_{i},\;$$

where $\alpha_{i}$ refers to the several variables in *C* and $\left(\frac{\partial S}{\partial \alpha_{i}}\right)$ refers to the respective conjugate variables. In the local-equilibrium theory, it is assumed that the entropy does only depend on the classical conserved variables. For instance, one has:

(47) $$dS=T^{-1}\left(dU+\tau_{ij}d\varepsilon_{ij}-\mu^{n}dn-\mu^{p}dp-\pi_{ij}da_{ij}\right),$$

where *T* is the absolute temperature, $\tau_{ij}$ is the stress tensor, and $\mu_{n}$, $\mu_{p}$, and $\pi_{ij}$ are the chemical potentials of n,p, and $a_{ij}$, respectively. These are intensive quantities, and their gradients produce fluxes of the respective conjugate quantities, namely, at equilibrium, the intensive quantities must be homogeneous. The functions $T^{-1}\left(U,\varepsilon_{ij},n,p,a_{ij}\right),\tau \left(U,\varepsilon_{ij},n,p,a_{ij}\right)$, and so on, giving the conjugates of the variable in terms of the variables, are called equations of state, and they determine many aspects of the behavior of the system (specific heat, thermal dilatation, elastic coefficients, chemical affinities, and so on).

The set of the dependent variables is, thus, given by:

(48) $$W=\left\{\tau_{ij},\mu^{n},\mu^{p},\pi_{ij},|U,S,\phi_{i},g^{n},g^{p},J_{i}^{n},J_{i}^{p},Q_{i},A_{ij},V_{ijk}\right\},$$

Then, to close the problem, i.e., to obtain the same number of quantities and independent equations, we must look for general constitutive equations in the form:$\;\;W=\tilde{W}\left(C\right),$ where both *C* and *W* are evaluated at the same point and time (more general possibilities are open if, for instance, the quantities *W* are given in terms of the history of *C* and of values of *C* in several points).

Among the various methods to analyze the entropy inequality, in [76], Liu’s theorem [192] was chosen, where all balance and evolution equations of the problem are considered as mathematical constraints for its physical validity. This theorem is used in rational thermodynamics and rational extended thermodynamics, which uses procedures different from that ones used in extended irreversible thermodynamics (which stems from CIT, classical irreversible thermodynamics, and exploits the dissipation inequality as in CIT [70,71,72,73,74]).

In practical situations, it is more useful using *T* as an independent variable than *U*, because temperature is much easier to measure and control than *U*. In such cases, it is convenient using the free energy density *F* as function, which is the Legendre transform of U(S,X) (with *X* standing for all the other variables of *U* except the entropy *S*, i.e., replacing *U* by $\frac{\partial S}{\partial U}$, as independent variable.

The free energy density F=F(C) is given by F=U−TS.

In terms of F(T,X), the second law of thermodynamics states that *F* can only decrease or keep constant (in strict terms, it states that the total *F* of the final equilibrium state must be lower or equal than the total *F* of the initial constrained equilibrium state). This requires that *F* can only reduce its value at any time, and assuming the mass density ρ is practically constant, neglecting the body force and the heat source distribution, the two main kinds of results are derived (see [41,42]):

(a) *The equations of state*, giving the partial derivatives of the free energy in terms of the respective conjugate thermodynamic variables:

$$\rho \frac{\partial F}{\partial E_{i}}=0,\;\rho \frac{\partial F}{\partial B_{i}}=0,\;\rho \frac{\partial F}{\partial \varepsilon_{ij}}=\tau_{ij},\;\frac{\partial F}{\partial n}=\mu^{n},\;\frac{\partial F}{\partial p}=\mu^{p},\;\frac{\partial F}{\partial T}=-S,$$

(49) $$\rho \frac{\partial F}{\partial a_{ij}}=\pi_{ij},\;\frac{\partial F}{\partial n_{,i}}=0,\;\frac{\partial F}{\partial p_{,i}}=0,\;\frac{\partial F}{\partial T_{,i}}=0,\;\frac{\partial F}{\partial a_{ij,k}}=0,$$

where $\mu^{n}$ and $\mu^{p}$ are the electrochemical potentials of *n* and *p*, respectively, and $\pi_{ij}$ is the tensorial chemical potential of the defect field $a_{ij}$ (defects contribute both to the internal energy and to the entropy of the system). In equilibrium thermodynamics, the equations of state play an essential role, for instance, in the determination of the phase diagram of the system, and on physical quantities such as the specific heat, the thermal expansion coefficient, the isothermal compressibility, and the elastic moduli. Thus, these equations contain and summarize much relevant information on the system.

Note that out of equilibrium, those functions may depend on the fluxes, but they do not directly depend on the gradients. In fact, close to a steady state, since in many occasions the fluxes are related to the gradients, the steady state values of the equations of state may formally depend on the gradients, after the fluxes have been written in terms of the latter.

A conceptual comment is of interest. If some of the fluxes, for Instance, the dislocation flux $V_{ijk}$, are no longer an independent quantity, but are proportional to the gradient of the conjugate variable $a_{ij,k}$ (namely $V_{ijk}=-Da_{ij,k}$), $a_{ij,k}$ may become a variable of *F*. Indeed, in this case, we may obtain that $\frac{\partial F}{\partial a_{ij,k}}$ is no longer zero, but it is $\frac{\partial F}{\partial a_{ij,k}}=\pi_{ij}\delta_{kr}$.

The *generalized affinities* conjugated to the respective fluxes of *n*, *p*, *U*, and $a_{ij}$ are as follows:

(50) $$\rho \frac{\partial F}{\partial j_{i}^{n}}=\Pi_{i}^{n},\;\rho \frac{\partial F}{\partial j_{i}^{p}}=\Pi_{i}^{p},\;\rho \frac{\partial F}{\partial q_{i}}=\Pi_{i}^{q},\;\rho \frac{\partial F}{\partial V_{ijk}}=\Pi_{ijk}^{\nu }.$$

In equilibrium thermodynamics, where the thermodynamic functions do not depend on the fluxes, there are no quantities analogous to the generalized affinities. These affinities are closely related to the inertia of the fluxes, as can be seen below.

An important statement related to the second law, expressed in terms of the stability of the equilibrium states (or nonequilibrium steady states), is that *F* must be a minimum with respect to variations in $X_{i}$ (the extensive quantities), namely:

(51) $${\left(\frac{\partial^{2}F}{\partial X_{i}\partial X_{j}}\right)}_{T,X_{k}}\geq 0,\;\mathrm{whereas}\;{\left(\frac{\partial^{2}F}{\partial T^{2}}\right)}_{X_{i}}\leq 0.$$

These relations imply, for instance, that the specific heat must be positive, or that:

$$\left(\frac{\partial \mu^{n}}{\partial n}\right)\geq 0,\;\;\;\left(\frac{\partial \mu^{n}}{\partial p}\right)\geq 0,\;\;\;\left(\frac{\partial \mu^{p}}{\partial n}\right)\geq 0,\;\;\;\left(\frac{\partial \mu^{p}}{\partial p}\right)\geq 0,\;$$

(52) $$\left(\frac{\partial \pi_{ij}}{\partial a_{kl}}\right)\geq 0,\;\;\;\left(\frac{\partial \pi_{ij}}{\partial p}\right)\geq 0,\;\;\;\left(\frac{\partial \pi_{ij}}{\partial n}\right)\geq 0,$$

and so on. For instance, mixtures or alloys of different materials (for instance, Si and Ge), may be mixed in a single homogeneous phase or may decompose in two different phases, depending on temperature, pressure, composition, and so on.

(b) The *residual inequality*, expressing the positive character of entropy production (or the negative character of the free energy production) is given by:

$$T\frac{\partial \phi_{k}}{\partial n}n_{,k}+T\frac{\partial \phi_{k}}{\partial p}p_{,k}+T\frac{\partial \phi_{k}}{\partial a_{ij}}a_{ij,k}+T\frac{\partial \phi_{k}}{\partial T}T_{,k}+$$

(53) $$+\left(j_{k}^{n}+j_{k}^{p}\right)E_{k}-\pi_{ij}A_{ij}-\Pi_{i}^{n}J_{i}^{n}-\Pi_{i}^{p}J_{i}^{p}-\Pi_{i}^{q}Q_{i}-\Pi_{ijk}^{\nu }V_{ijk}-\mu^{n}g^{n}-\mu^{p}g^{p}\geq 0.$$

Among other things, (53) restricts the possible forms of the entropy flux as well as the relations between the sources $J_{i}^{n},\;J_{i}^{p}$, $Q_{i}$, $V_{ijk}$ of the evolution equations of the fluxes and the generalized affinities $\Pi_{i}^{n},\;\Pi_{i}^{p},\;\Pi_{i}^{q},\;\Pi_{ijk}^{\nu }$. In more explicit terms, this inequality restricts the form of the transport equations.

The entropy flux $\phi_{k}$ is as follows:

(54) $$\phi_{k}=\frac{1}{T}\left(q_{k}-\mu^{n}j_{k}^{n}-\mu^{p}j_{k}^{p}-\pi_{ij}V_{ijk}\right).$$

where the first three terms are the classical expression for the entropy flux in semiconductors: they have the form of the internal energy flux $q_{k}$ times the thermodynamic conjugate to the internal energy with respect to the entropy (i.e., $\;T^{-1}$), plus the electron and hole fluxes $\left(j_{k}^{n},j_{k}^{p}\right)$ times the respective conjugates to electron charge and hole charge $\left(-\mu^{n}/T,-\mu^{p}/T\right)$. The last term is the contribution of the defects (line defects), and it has the form of the defects flux $\left(V_{ijk}\right)$ times the thermodynamic conjugate to the defect tensor $\left(-\pi_{ij}/T\right)$. In the case when $V_{ijk}=-Da_{ij,k}$, the term in $\left(V_{ijk}\right)$ in (54) must be accordingly replaced.

On some occasions, working with (53) is easier and more direct if instead of using the basic variables $n,p,a_{ij}$, etc., one uses their conjugate quantities $\mu^{n},\mu^{p},\pi_{ij}$ and one requires the fluxes to be linear function of the thermodynamic forces (namely, the gradients of the mentioned conjugate quantities) [55,152,153]. In this case the constitutive equations are as follows:

$$q_{k}=L_{qq}T_{,k}^{-1}+L_{qp}\left(-\mu_{,k}^{p}\right)+L_{qn}\left(-\mu_{,k}^{n}\right)+L_{qa}\left(-\pi_{ij,k}\right),$$

$$j_{k}^{n}=L_{nq}T_{,k}^{-1}+L_{np}\left(-\mu_{,k}^{p}\right)+L_{nn}\left(-\mu_{,k}^{n}\right)+L_{na}\left(-\pi_{ij,k}\right),$$

$$j_{k}^{p}=L_{pq}T_{,k}^{-1}+L_{pp}\left(-\mu_{,k}^{p}\right)+L_{pn}\left(-\mu_{,k}^{n}\right)+L_{pa}\left(-\pi_{ij,k}\right),$$

(55) $$V_{ijk}=L_{aq}T_{,k}^{-1}+L_{ap}\left(-\mu_{,k}^{p}\right)+L_{an}\left(-\mu_{,k}^{n}\right)+L_{aa}\left(-\pi_{ij,k}\right),$$

and the positive character of (53) is insured if one requires that the matrix of the phenomenological coefficients **L**(related to the transport coefficients) is definitely positive. Furthermore, according to the Onsager reciprocity relations, the matrix *L* is symmetrical. It turns out from (49) that the free energy *F* is a function of:

(56) $$F=F(\varepsilon_{ij},n,p,T,a_{ij},j_{i}^{n},j_{i}^{p},q_{i},V_{ijk}),$$

namely, it does not depend on the gradients of $n,p,T,a_{ij},E_{i}$ nor $B_{i}$. This means that in this formalism nonlocal effects are described by the fluxes in the rate Equation (44). The two features we have mentioned restrict the equations of state (thermodynamic stability) and the transport Equations (positive entropy production).

## 5. Constitutive Theory and Rate Equations for Charges, Heat, and Defect Field

Here, we expand the free energy (56) up to second-order approximation in the deviations of the independent variables around the local equilibrium state [41,42]. Such a state is characterized by the equilibrium form of the thermodynamic functions, but corresponds to the local values of the classical variables. Taking the local-equilibrium state as a reference state is typical of the classical irreversible thermodynamics, and it leads to satisfactory results provided the perturbations are not too fast nor too steep. To enlarge the domain of validity of thermodynamics, the fluxes (or internal variables, or other nonequilibrium variables) may be incorporated. Thus, we assume that up the second order, the most general admissible form of $F-F_{eq}$ for anisotropic systems in terms of the fluxes $q_{i},j_{i}^{n},j_{i}^{p}$ is as follows:

$$F\left(\varepsilon_{ij},n,p,T,a_{ij},j_{i}^{n},j_{i}^{p},q_{i}\right)-F_{eq}\left(\varepsilon_{ij},n,p,T,a_{ij}\right)=\frac{1}{2\rho }\lambda_{ij}^{qq}q_{i}q_{j}+\frac{1}{2\rho }\lambda_{ij}^{j^{n}j^{n}}j_{i}^{n}j_{j}^{n}+$$

(57) $$\frac{1}{2\rho }\lambda_{ij}^{j^{p}j^{p}}j_{i}^{p}j_{j}^{p}+\frac{1}{\rho }\lambda_{ij}^{j^{n}q}j_{i}^{n}q_{j}+\frac{1}{\rho }\lambda_{ij}^{j^{p}q}j_{i}^{p}q_{j}+\frac{1}{\rho }\lambda_{ij}^{j^{n}j^{p}}j_{i}^{n}j_{j}^{p}-S_{0}\left(\theta \right).$$

where we have taken into account the physical dimensions of the quantities (the introduction of minus sign in the last term comes from physical reasons) and the invariance of *F* under time reversal (so that the terms containing the fluxes at first order are null). In extended thermodynamics, the coefficients related to the fluxes (namely $\lambda_{ij}^{qq}$, $\lambda_{ij}^{j^{n}j^{n}}$, and so on) are proportional to the relaxation times of the fluxes, and vanish when such relaxation times are negligible. Since *F* must be minimum at equilibrium, the matrices λ in (57) must be definitely positive.

Some phenomenological coefficients, which are supposed to depend on the local-equilibrium variables but not on the fluxes, satisfy the following symmetry relations:

(58) $$\lambda_{ij}^{qq}=\lambda_{ji}^{qq},\;\lambda_{ij}^{j^{n}j^{n}}=\lambda_{ji}^{j^{n}j^{n}},\;\lambda_{ij}^{j^{p}j^{p}}=\lambda_{ji}^{j^{p}j^{p}},\;\lambda_{ij}^{j^{n}q}=\lambda_{ji}^{j^{n}q},\;\lambda_{ij}^{j^{p}q}=\lambda_{ji}^{j^{p}q},\;\lambda_{ij}^{j^{n}j^{p}}=\lambda_{ji}^{j^{n}j^{p}}.$$

The particular values of these coefficients depend on the structure of the anisotropic systems. For instance, in the superlattice of Figure 1, direction *x* perpendicular to the interfaces is clearly different than directions *y* and *z*. In general terms, one could describe the geometry of the superlattice by means of a tensor, and use it in the construction of some additional terms of the free energy *F*.

Finally, it is interesting to stress [73] the stability criterion, establishing that *F* tends to a minimum. This thermodynamic stability is a corollary of the entropy principle, implying that in a thermodynamic process, the entropy production is nonnegative so that the entropy tends to a maximum. From this thermodynamic stability, it is possible to obtain some restrictions for the phenomenological coefficients in (57).

### 5.1. Constitutive Relations: Equations of State

According to the local-equilibrium hypothesis, we assume that the local-equilibrium form of the equations of state for $\tau_{ij},S,\mu^{n},\mu^{p}$, and $\pi_{ij}$ are known. Nonequilibrium contributions from the fluxes are obtained from the right-hand side of (57). Such equations are in general nonlinear, but they may be linearized around the local-equilibrium state in order to study the evolution of small perturbations.

From these expressions, the contribution of the fluxes to the equations of state may be obtained. From (57) and (49), we obtain the following *constitutive relations* (or equations of state) for the conjugate thermodynamic variables $\tau_{ij},S,\mu^{n},\mu^{p}$, and $\pi_{ij}$ defined in (49):

(59) $$\tau_{ij}=\tau_{ijeq}+\frac{\partial }{\partial \varepsilon_{ij}}\left[\frac{1}{2\rho }\lambda_{ij}^{qq}q_{i}q_{j}+\ldots \right],$$

(60) $$S=S_{eq}-\frac{\partial }{\partial T}\left[\frac{1}{2\rho }\lambda_{ij}^{qq}q_{i}q_{j}+\ldots \right],$$

(61) $$\mu^{n}=\mu_{eq}^{n}+\frac{\partial }{\partial n}\left[\frac{1}{2\rho }\lambda_{ij}^{qq}q_{i}q_{j}+\ldots \right],$$

(62) $$\mu^{p}=\mu_{eq}^{p}+\frac{\partial }{\partial p}\left[\frac{1}{2\rho }\lambda_{ij}^{qq}q_{i}q_{j}+\ldots \right],$$

(63) $$\pi_{ij}=\pi_{ijeq}+\frac{\partial }{\partial a_{ij}}\left[\frac{1}{2\rho }\lambda_{ij}^{qq}q_{i}q_{j}+\ldots \right],$$

where $\frac{1}{2\rho }\lambda_{ij}^{qq}q_{i}q_{j}+\ldots$ means the flux-dependent terms on the right-hand side of (57). Note that in such terms, the dependence on $T,n,p,a_{ij}$, and $\varepsilon_{ij}$ appears through the coefficients $\lambda_{ij}^{qq},\lambda_{ij}^{J^{n}J^{n}}$, and so on.

The flux contributions to (59)–(63) may be relevant for high values of the fluxes, and may lead to modifications of the phase diagram, leading, for instance, to an instability of the system related to changes in the signs of (52) (i.e., two materials which are miscible with each other at equilibrium may separate under the action of a sufficiently high flux). For instance, it could be that $\frac{\partial \mu^{n}}{\partial n}>0$, a stability in local-equilibrium condition, but $\frac{\partial \mu^{n}}{\partial n}$ could become negative (instability condition) for $q_{i}$ or $j_{i}^{n}$, or $j_{i}^{p}$ sufficiently high.

From (50) and (57) follow for the generalized affinities, conjugated to the fluxes, the following linearized expressions:

(64) $$\Pi_{i}^{q}=\lambda_{ij}^{qq}q_{j}+\lambda_{ij}^{qj^{n}}j_{j}^{n}+\lambda_{ij}^{qj^{p}}j_{j}^{p},$$

(65) $$\Pi_{i}^{n}=\lambda_{ij}^{j^{n}q}q_{j}+\lambda_{ij}^{j^{n}j^{n}}j_{j}^{n}+\lambda_{ij}^{j^{n}j^{p}}j_{j}^{p},\;\;\Pi_{i}^{p}=\lambda_{ij}^{j^{p}q}q_{j}+\lambda_{ij}^{j^{p}j^{p}}j_{j}^{p}+\lambda_{ij}^{j^{n}j^{p}}j_{j}^{n}.$$

In the case that the flux of dislocations, $V_{ijk}$ is considered an independent variable, and a further equation would appear besides (64) and (65). According to EIT (extended irreversible thermodynamics) [55,56], the coefficients $\lambda_{ij}^{qq}$, $\;\lambda_{ij}^{j^{n}j^{n}}$ and $\lambda_{ij}^{j^{p}j^{p}}$ are proportional to the relaxation times of $j^{n}$, $j^{p}$, and q, in isotropic systems $\lambda_{ij}^{qq}=\tau^{q}\delta_{ij}$, $\lambda_{ij}^{j^{n}j^{n}}=\tau^{j^{n}}\delta_{ij}$, and $\lambda_{ij}^{j^{p}j^{p}}=\tau^{j^{p}}\delta_{ij}$. These equations are necessary because, as we have commented above, the residual inequality (55) sets a strong connection between the source terms in (44) and the generalized affinities given by (64), (65). Furthermore, relations (64), (65) are helpful to understand the physical meaning of the respective physical coefficients, appearing in the free energy (57).

### 5.2. Rate Equations for the Fluxes and the Defects

The residual inequality (55) imposes some relations among the source terms $J_{i}^{n}\left(C\right)$, $J_{i}^{p}\left(C\right)$, $J_{i}^{q}\left(C\right)$, $A_{ij}\left(C\right)$, and their conjugates (64), (65), and (63). Here, the source terms $J_{i}^{n}\left(C\right)$, $J_{i}^{p}\left(C\right)$, $J_{i}^{q}\left(C\right)$, and $A_{ij}\left(C\right)$ for small perturbations of the variables are expressed as linear polynomials, with constant coefficients, in terms of the independent variables in the following form [41,42]:

$${\dot{q}}_{i}=\chi_{ijk}^{1}\varepsilon_{jk}-\chi_{ij}^{2}E_{j}+\chi_{ijk}^{3}\alpha_{jk}+\chi_{ij}^{4}j_{j}^{n}+\chi_{ij}^{5}j_{j}^{p}-$$

(66) $$\chi_{ij}^{6}q_{j}-\chi_{ij}^{7}\theta_{,j}+\chi_{ijkl}^{8}\alpha_{jk,l}+\chi_{ij}^{9}N_{,j}+\chi_{ij}^{10}P_{,j},$$

$${\dot{j}}_{i}^{n}=\eta_{ijk}^{1}\varepsilon_{jk}+\eta_{ij}^{2}E_{j}+\eta_{ijk}^{3}\alpha_{jk}-\eta_{ij}^{4}j_{j}^{n}+\eta_{ij}^{5}j_{j}^{p}+$$

(67) $$\eta_{ij}^{6}q_{j}-\eta_{ij}^{7}\theta_{,j}+\eta_{ijkl}^{8}\alpha_{jk,l}+\eta_{ij}^{9}N_{,j}+\eta_{ij}^{10}P_{,j},$$

$${\dot{j}}_{i}^{p}=\omega_{ijk}^{1}\varepsilon_{jk}+\omega_{ij}^{2}E_{j}+\omega_{ijk}^{3}\alpha_{jk}+\omega_{ij}^{4}j_{j}^{n}-\omega_{ij}^{5}j_{j}^{p}+$$

(68) $$\omega_{ij}^{6}q_{j}-\omega_{ij}^{7}\theta_{,j}+\omega_{ijkl}^{8}\alpha_{jk,l}+\omega_{ij}^{9}+N_{,j}+\omega_{ij}^{10}P_{,j},$$

$${\dot{\alpha }}_{ij}+D^{\prime }a_{ij,kk}=\beta_{ijkl}^{1}\varepsilon_{kl}+\beta_{ijk}^{2}E_{k}+\beta_{ij}^{3}N+\beta_{ij}^{4}P+\beta_{ij}^{5}\theta -\beta_{ijkl}^{6}\alpha_{kl}+$$

(69) $$\beta_{ijk}^{7}j_{k}^{n}+\beta_{ijk}^{8}j_{k}^{p}+\beta_{ijk}^{9}q_{k}-\beta_{ijk}^{10}\theta_{,k}+\beta_{ijklm}^{11}\alpha_{kl,m}+\beta_{ijk}^{12}N_{,k}+\beta_{ijk}^{13}P_{,k}.$$

Note that alternative expressions for $\dot{q},{\dot{j}}_{i}^{n},{\dot{j}}_{i}^{p},{\dot{\alpha }}_{ij}$ may be given in terms of the gradients of the conjugate variables, instead of in terms of independent variables. It is useful to have this point in mind when one wants to compare these expressions with those of classical irreversible thermodynamics. The rate Equations (66)–(69) allow finite speeds for the field disturbances and describe fast phenomena, whose relaxation time is comparable or higher than the relaxation time of the media under consideration.

In this paper, we are especially interested in two kinds of anisotropic systems, namely, parallel multilayers and concentric multilayers. In the multilayer systems, there is a privileged axis *z* perpendicular to the layers. Thus, the several tensors appearing in (66)–(69) *do have* the form (1), namely:

(70) $$\lambda =\left|\left|\begin{array}{ccc} \lambda_{xx}\left(z\right) & \lambda_{yx}\left(z\right) & 0\\ \lambda_{xy}\left(z\right) & \lambda_{yy}\left(z\right) & 0\\ 0 & 0 & \lambda_{zz}\left(z\right) \end{array}\right|\right|.$$

for all the different tensors. Then, (66)–(69) can be rewritten in the following form:

(71) $$\tau^{j^{n}}\cdot {\dot{j}}^{n}+j^{n}=\Sigma^{n}\cdot \tilde{E}-\chi^{n}\cdot \nabla \theta +\eta^{1}\cdot q+\eta^{2}\cdot j^{p}+\eta^{3}\cdot \nabla \mathrm{Tr}\alpha ,$$

(72) $$\tau^{j^{p}}\cdot {\dot{j}}^{p}+j_{i}^{p}=\Sigma^{p}\cdot \tilde{E}-\chi^{p}\cdot \nabla \theta +\omega^{1}\cdot q+\omega^{2}\cdot j^{n}+\omega^{3}\cdot \nabla \mathrm{Tr}\alpha .$$

(73) $$\tau^{q}\cdot \dot{q}+q=\Sigma^{q}\cdot \tilde{E}-\chi^{q}\cdot \nabla \theta +\lambda^{1}\cdot j^{n}+\lambda^{2}\cdot j^{p}+\lambda^{3}\cdot \nabla \mathrm{Tr}\alpha .$$

(74) $$\tau^{\alpha }\cdot \dot{\alpha }+\alpha =-D^{\prime }\nabla^{2}\alpha -\lambda^{q}\cdot \tilde{E}-\lambda^{\alpha q}\cdot q-\lambda^{\alpha p}\cdot j^{p}-\lambda^{\alpha n}\cdot j^{n},$$

where $\tau^{\alpha },\;\tau^{j^{n}},\;\tau^{j^{p}},\;\tau^{q}\;$ are the relaxation time tensors of the fields $\alpha ,\;j^{n},\;j^{p}$, and q are, respectively, the phenomenological tensors, which are constant, $\Sigma^{n},\;\Sigma^{p}$ are conductivity tensors, $\Sigma^{q}$ is the Peltier tensor, $\chi^{n},\;\chi^{p}$ are Seebeck tensors, and $\chi^{q}$ is the heat conductivity tensor, while the other phenomenological tensors describe interactions between the different fields.

The rate Equation (73) for the heat flux generalizes Vernotte–Cattaneo relation. In it $\chi_{ij}^{2}$ is related to the Peltier constants and $\chi_{ij}^{7}$ is related to the heat conductivity coefficients, $\chi_{ij}^{6}=\frac{1}{\tau^{q}}$, where $\tau^{q}$ is the relaxation time of the heat flux. In (67) and (68) $\eta_{ij}^{2}$, $\omega_{ij}^{2}$ is constant and related to the electric conductibility coefficients and $\eta_{ij}^{6},$ and $\omega_{ij}^{6}$ are related to the Seebeck coefficients. The minus signs are due to physical reasons. Analogously, in (67) and (68), we have $\eta_{ij}^{4}=\frac{1}{\tau^{n}}$, $\eta_{ij}^{2}=\frac{\sigma^{n}}{\tau^{n}}$, $\omega_{ij}^{5}=\frac{1}{\tau^{p}}$, and $\omega_{ij}^{2}=\frac{\sigma^{p}}{\tau^{p}}$, where $\sigma^{n}$ and $\sigma^{p}$ are the electrical conductibility of electrons and holes and $\tau^{n}$ and $\tau^{p}$ are the relaxation times of the electrons current and holes current, and $\eta_{ij}^{6}=\frac{\pi^{n}}{\tau^{n}}$, $\omega_{ij}^{6}=\frac{\pi^{p}}{\tau^{p}}$, where $\pi^{n}$ and $\pi^{p}$ are the electrons and holes contributions to the Peltier effect. The last term in (71)–(73) describes nonlocal effects, as it relates to the rate equations to inhomogeneities in the system. In [193], numerical data regarding the phenomenological coefficients are given.

### 5.3. Conditions at the Interfaces

In multilayer systems (such as superlattices, discontinuously graded systems, or metamaterials) the conditions at the interfaces play a relevant role. In them, there appear discontinuities of the temperature θ, and the chemical potentials $\mu^{n}$, $\mu^{p},$ and $\pi_{ij}$. Here, we assume the fluxes go in the *x* direction, whereas the interfaces correspond to yz planes. In this case, the conditions at the interfaces are as follows (see [42]):

(75) $$\theta_{A}-\theta_{B}=R_{AB}^{q}q_{x},\;\;\pi_{A}^{yz}-\pi_{B}^{yz}=R_{AB}^{a}V_{zyx},\;\;\mu_{A}^{n}-\mu_{B}^{n}=R_{AB}^{n}j_{x}^{n},\;\;\mu_{A}^{p}-\mu_{B}^{p}=R_{AB}^{p}j_{x}^{p}.$$

where $R_{AB}^{q}$, $R_{AB}^{n}$, $R_{AB}^{p}$, and $R_{AB}^{a}$ refer to the respective boundary resistances related to heat, charges, and dislocations diffusion orthogonal to the interface.

In more general terms, one would have also coupling terms relating the discontinuities in $T,\mu^{n},\mu^{p}$, and so on to all the fluxes $j^{n},j^{p},q$, and so on as follows:

(76) $$R_{AB}^{q}q_{x}=\theta_{A}-\theta_{B}+R_{AB}^{\prime qn}\left(\mu_{A}^{n}-\mu_{B}^{n}\right)+R_{AB}^{\prime qp}\left(\mu_{A}^{p}-\mu_{B}^{p}\right),$$

(77) $$R_{AB}^{n}j_{x}^{n}=\mu_{A}^{n}-\mu_{B}^{n}+R_{AB}^{\prime np}\left(\mu_{A}^{p}-\mu_{B}^{p}\right)+R_{AB}^{\prime n\theta }\left(\theta_{A}-\theta_{B}\right),$$

(78) $$R_{AB}^{p}j_{x}^{p}=\mu_{A}^{p}-\mu_{B}^{p}+R_{AB}^{\prime pn}\left(\mu_{A}^{n}-\mu_{B}^{n}\right)+R_{AB}^{\prime p\theta }\left(\theta_{A}-\theta_{B}\right),$$

where $R_{AB}^{\prime }$ refers to the corresponding coupling coefficients. The form of the thermal resistance $R_{AB}^{q}$ is the subject of much microscopic reasearch. One tries to express $R_{AB}^{q}$ in terms of properties of the materials A and B on both sides of the interface. The two simplest models are the so-called acoustic mismatch modem (AMM) and diffusion mismatch model (DMM). In the first model, $R_{AB}^{q}$ refers to differences in the phonon speed and in the density of materials A and B, namely, in terms of $\rho_{A}V_{A}$ and $\rho_{B}V_{B}$. In the second model, one relates them to differences in the thermal conductivity $\lambda_{A}$ and $\lambda_{B}$. These two models do not take into account microscopic details, such as the cell-size mismatch or the frequency mismatch in both materials. Terms such as this one are considered in microscopic techniques based in the Boltzmann transport equation.

## 6. Systems with Mobile Defects—Some New Results and Possible Applications

In this section, we present some new results and possible applications coming from the present formalism, in which we combine the influence of stress, defects, and nanostructures on the transport coefficients. From the more general perspective gained in Section 3 and Section 4, we examine some additional aspects of the problems presented in Section 2.

We consider some possible effects in systems with mobile defects, going beyond the influence of static defects considered in Section 2.2.1 and Section 2.2.2. We consider the minimum set of equations to outline the wide range of possibilities in the combination of phenomena. Fourier’s law for the heat flux is used, namely:

(79) q=−λ(T,c)∇T,

and an equation for the flux of defects

(80) $$J=-D\nabla c+\alpha_{1}cq+\alpha_{2}cq_{e}E,$$

where *D* is the diffusion coefficient of defects, and the terms in $\alpha_{1}cq$ and $\alpha_{2}cq_{e}E$ representing a drift of defects under the action of a heat flux q [194,195,196,197] or, in the case that the defects are electrically charged (with charge $q_{e}$), the drift under an applied electric field E. This simple set of equations leads to interesting physical effects that we examine in the next subsections. More general impressions, including a contribution of the flux of defects to the heat flux, would be also be possible. Here, we aim to stress that an indirect coupling between q and J may be established through the dependence of the thermal conductivity on the defect concentration.

### 6.1. A Mathematical Model for a Thermal Transistor Based on Mobile Defects

In Section 2.3.2, we have illustrated the role of defects in achieving thermal transistors Here, we consider mobile defects instead of the static defects considered in Section 2.2.2 [45]. To our knowledge, no such devices with mobile defects are yet used, and this section is a theoretical proposal. This proposal is restricted, in practice, to steady-state situations, because the motion of defects in solids is usually slow (unless the defects are very small and the atomic separation in the lattice is wider than the size of the defects); on the other hand, the proposal allows to introduce and control new kinds of nonlinearities of the coefficients with respect to the heat flux, and this could be useful for future applications.

**Figure 15:**
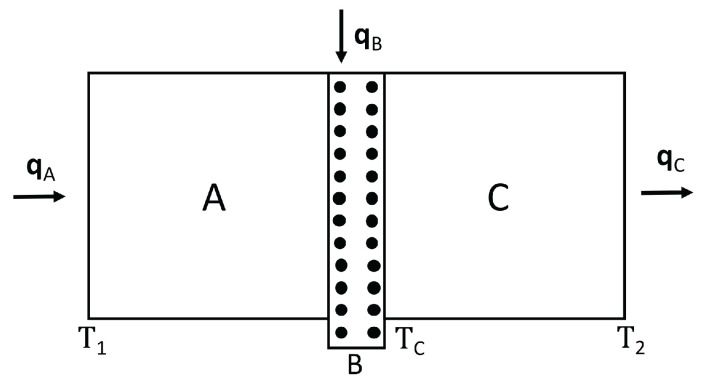
Heat transistor system, based on mobile defects driven by a heat flux considered in this Section.

The system is sketched in Figure 15. In our proposal, a thin layer of a material B containing mobile defects able to move along the layer, is sandwiched between two pieces of materials A and C (in Section 2.2.2, A and C were assumed to have different concentrations of static defects; here, they do not need to have defects, as we focus our attention on the mobile defects in the sandwiched layer between A and C). Part A is traversed by a heat flux $q_{A}$, perpendicular to the B thin layer, and along the layer B, a heat flux $q_{B}$ is injected. The total heat supplied to A and B per unit time flows out of the system through part C.

The heat flux $q_{B}$ produces a drift of defects along the direction of $q_{B}$, and thus, the heat flux *B* carries out a number of defects from *B*, and as a consequence, it increases the thermal conductivity of layer *B*. To make the removal of such defects easier while $q_{B}$ is flowing, we make layer B slightly longer than the width of sections A and C, so that a fraction of the driven defects may accumulate in the extra region, thus avoiding that they contribute to the transversal thermal resistance of the layer.

As said in Section 3.2, in order for this system to be considered as a transistor, it is necessary that: $\frac{\partial q_{C}}{\partial q_{B}}>1.$ This implies that the variations in $q_{C}$ are amplified through variations of $q_{B}$.

The equations describing the fluxes $q_{A}$ and $q_{C}$ between $T_{1}$ and $T_{C}$ (the temperature of the layer) and between $T_{C}$ and $T_{2}$, respectively, are as follows:

(81) $$T_{1}-T_{C}=\left[\frac{L_{A}}{\lambda_{A}}+\frac{L_{B}}{\lambda_{B}\left(q_{B}\right)}\right]q_{A}\equiv R_{AB}\left(q_{B}\right)q_{A},\;T_{C}-T_{2}=\frac{L_{C}}{\lambda_{C}}q_{C}\equiv R_{C}q_{C},$$

where $R_{A}$ and $R_{C}$ are the thermal resistances of A+B and of *C*, respectively. In this simple formulation, we neglect the thermal resistances at the interfaces AB and BC, but there is no difficulty in incorporating them in a more accurate but more cumbersome analysis. Equation (81) follows from the direct application of Fourier’s law. A difference from the situation considered in Section 2.2.2 here the thermal conductivity of layer B depends explicitly of the heat flux $q_{B}$, because it produces a redistribution of defects.

The value of $T_{C}$ is found from the steady-state condition $q_{C}=q_{A}+q_{B}$. This implies that:

(82) $$R_{C}^{-1}\left[T_{C}-T_{2}\right]=R_{AB}^{-1}\left[T_{1}-T_{C}\right]+q_{B}.$$

From here, one obtains for $T_{C}$ as a function of $q_{B}$:

(83) $$T_{C}=\frac{R_{AB}^{-1}T_{1}+R_{C}^{-1}T_{2}+q_{B}}{R_{AB}^{-1}+R_{C}^{-1}}.$$

Introducing this expression for $T_{C}$ into Equation (81)${}_{1}$, one obtains:

(84) $$q_{A}=R_{AB}^{-1}T_{1}-\frac{R_{AB}^{-1}}{R_{AB}^{-1}+R_{C}^{-1}}\left[R_{AB}^{-1}T_{1}+R_{C}^{-1}T_{2}+q_{B}\right].$$

From here, the relation $\frac{\partial q_{A}}{\partial q_{B}}$ may be obtained taking into account that $R_{AB}^{-1}$ depends on $q_{B}$, since an increase in $q_{B}$ produces a decrease of $R_{AB}$. From here, we obtain the amplification factor:

$$\frac{\partial q_{C}}{\partial q_{B}}=1+\frac{\partial q_{A}}{\partial q_{B}}=1-\frac{R_{AB}^{-1}}{R_{AB}^{-1}+R_{C}^{-1}}\left[1+\Gamma_{AB}T_{1}\right]+$$

(85) $$+\Gamma_{AB}T_{1}-\frac{R_{AB}^{-1}T_{1}+R_{C}^{-1}T_{2}+q_{B}}{{\left[R_{AB}^{-1}+R_{C}^{-1}\right]}^{2}}\Gamma_{AB}R_{C}^{-1},$$

where $\Gamma_{AB}$ stands for $\Gamma_{AB}\equiv \frac{\partial R_{AB}^{-1}}{\partial q_{B}}$. If $\Gamma_{AB}=0$, (85) reduces to:

(86) $$\frac{\partial q_{C}}{\partial q_{B}}=\frac{R_{C}^{-1}}{R_{AB}^{-1}+R_{C}^{-1}}<1,$$

and the device is not a transistor. In our case, since $R_{AB}=R_{A}+R_{B}\left(q_{B}\right)$, and $R_{B}$ (namely, $\frac{L_{B}}{\lambda_{B}\left(q_{B}\right)}$) decreases with an increase of $q_{B}$, one has $\frac{\partial R_{AB}}{\partial q_{B}}<0$, and thus, $\Gamma_{AB}=-R_{AB}^{-2}\frac{\partial R_{AB}}{\partial q_{B}}>0$. From (85), it follows that the amplification factor $\;\frac{\partial q_{C}}{\partial q_{B}}\;$ will be higher than 1 if:

(87) $$\Gamma_{AB}>\frac{R_{AB}^{-1}}{R_{C}^{-1}}+\frac{1}{T_{1}-T_{2}}.$$

In order to model how $q_{B}$ reduces the total concentration of defects in the layer *B* (and therefore, increasing the transversal thermal conductivity in this layer), we assume that the flux of defects is given by (80) (with E=0):

(88) $$J_{def}=-D\nabla c+c\alpha_{1}q,$$

where $\alpha_{1}q$ shows the drift velocity of defects under the presence of a heat flow (this could be considered, for instance, as a phonon drag of defects or as an analogous of thermophoretic effect).

Thus, in steady state with $J_{def}=0$, we have from (88):

(89) $$c\left(z\right)=c\left(0\right)exp\left[\frac{\alpha_{1}q}{D}z\right]+\mathrm{constant},$$

where the constant is determined by the boundary conditions. For q=0, the concentration of defects is homogeneous in the layer *B*. The higher *q* is, the shorter the characteristic length $l\equiv D/\alpha_{1}q$, where the defects become concentrated.

**Figure 16:**
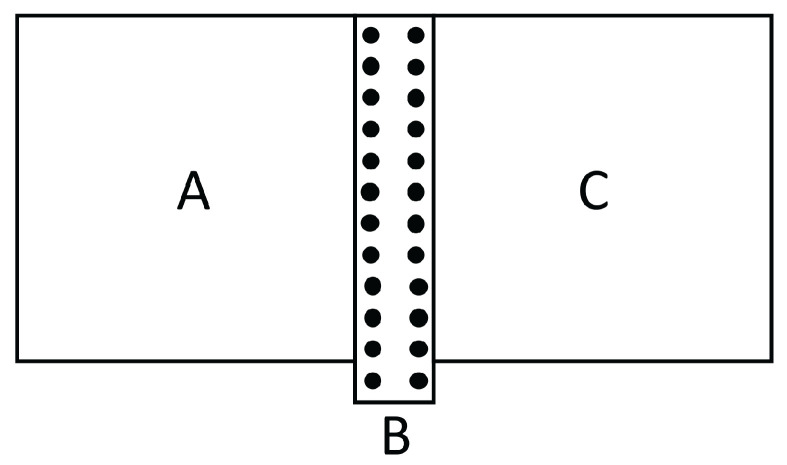
Homogeneous distribution of defects in the layer B when q=0.

**Figure 17:**
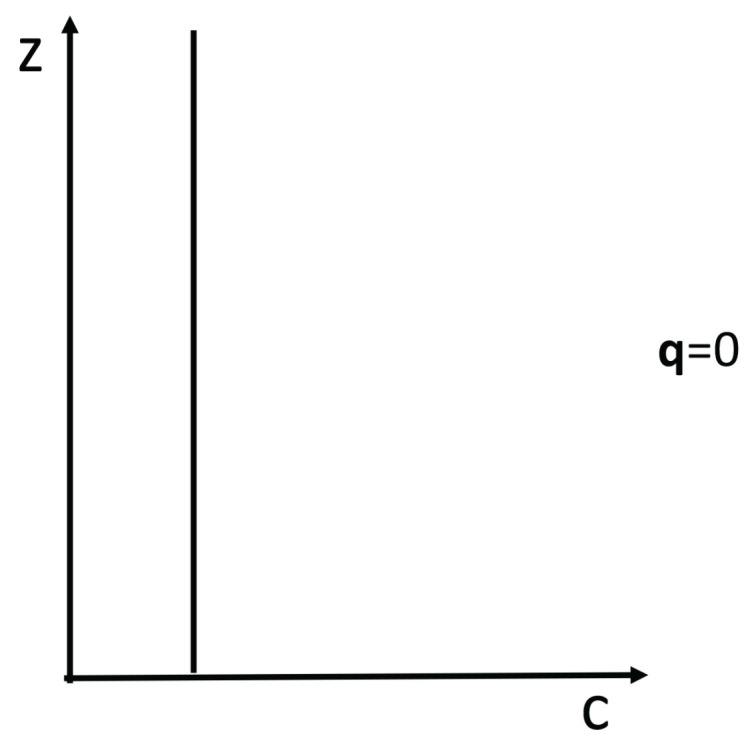
Representation of defects’ concentration when q=0.

**Figure 18:**
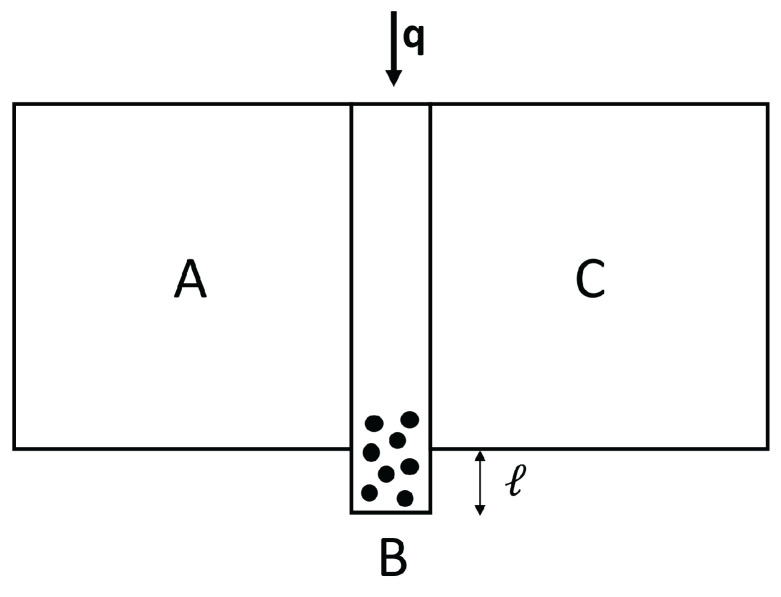
Inhomogeneous distribution of defects in the layer B when q≠0.

**Figure 19:**
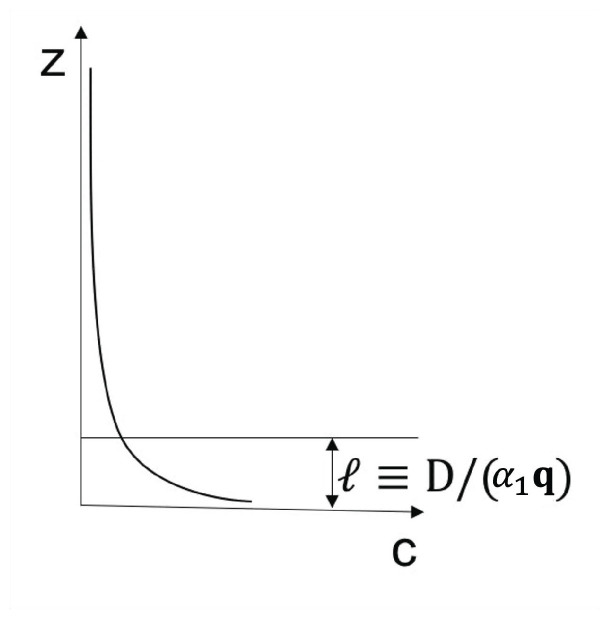
Representation of defects’ concentration when q≠0.

In our model, we propose that the layer B has a length wider than the width of A and C (see Figure 16, Figure 17, Figure 18 and Figure 19). In this way, a fraction of defects will accumulate in this extra zone, and will go out from the region where they reduce the heat flux. The effective concentration of defects in the zone of the heat flow will be reduced and the reduction of thermal resistance for a given heat flux will be more effective the longer the additional length *d* of the layer.

For the sake of a simple illustration, assume that:

(90) $$\lambda_{B}\left(T,c\right)=\frac{\lambda_{B0}\left(T\right)}{1+\beta T_{C}}\approx \lambda_{B0}\left(T\right)\left[1-\beta T_{C}\right].$$

We will have:

(91) $$\frac{\partial R_{B}}{\partial q}=\frac{L_{B}}{\lambda_{B0}}\beta T\frac{\partial c}{\partial q}<0,\;\;i.e.,\;\;\Gamma_{AB}=-\frac{L_{B}}{\lambda_{B0}R_{AB}^{2}}\beta T\frac{\partial c}{\partial q}.$$

In view of relation (87), this means that the present model will work as a thermal transistor provided that $-\frac{\partial c}{\partial q}⩾\frac{\lambda_{B0}}{L_{B}}\frac{R_{AB}R_{C}}{\beta T_{C}\left(T_{1}-T_{C}\right)}.$

To have this behavior, it will be convenient that $\alpha_{1}$ is high, *D* is low, and the additional depth *d* of layer *B* is relatively long (see (89) and (92), (93) in Section 6.2). In the first order in *q*, the effective concentration c(q) in the layer B will be $c\left(q\right)=\frac{c_{0}}{1+\frac{\alpha_{1}q}{2D}L_{B}}$.

### 6.2. Steady-State Nonlinear Heat Transport in Superlattices with Mobile Defects

In Section 6.1, we have considered the motion of defects in the sandwiched layer between *A* and *C*. Here, we consider a system composed of *A* and *B* (or a superlattice formed by a repetitive sequence of *A* and *B*), and the heat perpendicular to their interface. We assume that the latter cannot be crossed by defects, in such a way that J=0. As in (89) (see also (88)), we will have an exponential distribution of defects with a maximum at the interface. The value of c(0) in (89) may be obtained in terms of the original homogeneous concentration of defects *c* in the absence of the heat flux, by considering the conservation of the total number of defects, namely:

(92) $$c\;L=\int_{0}^{L}c\left(x\right)dx,\;i.e.,\;c\;L=c\left(0\right)\frac{D}{\alpha q}\left[exp\left(\frac{\alpha_{1}qL}{D}\right)-1\right].$$

Up to the first order in $\frac{\alpha_{1}qL}{D}$, the condition (92) leads to

$$\;\;\;c\left(0\right)=\frac{c}{1+\frac{1}{2}\frac{\alpha_{1}qL}{D}}.$$

**Figure 20:**
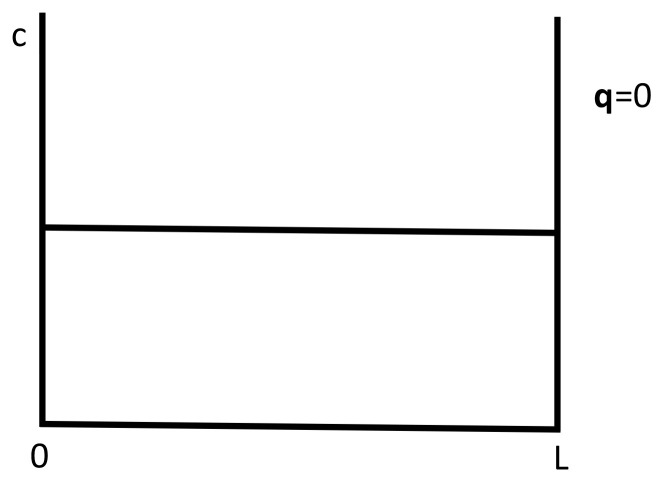
Constant concentration of defects when q=0.

**Figure 21:**
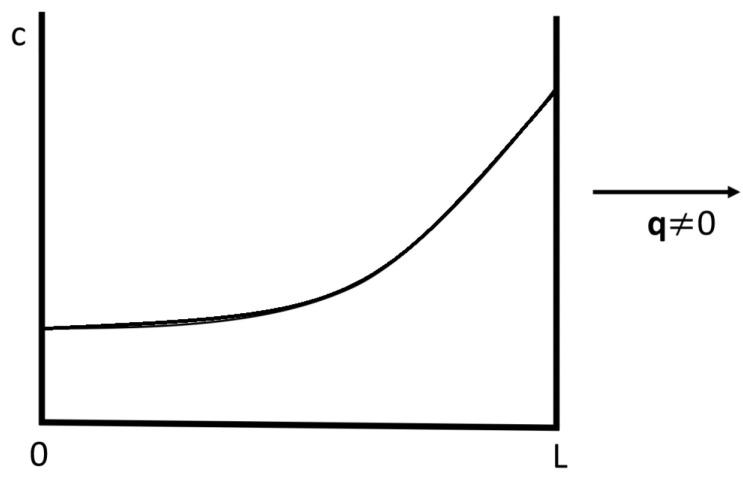
Concentration of defects when q≠0.

In Figure 20 and Figure 21, the concentration of defects is plotted when q=0 and q≠0, respectively.

Then, the thermal resistance of system *A* will be:

(93) $$\mathrm{Thermalresistance}=\int_{0}^{L}\frac{dx}{\lambda (x)}=\int_{0}^{L}\frac{1+\beta_{1}T^{b}c^{a}\left(x\right)}{\lambda_{0}\left(T\right)}dx.$$

**Figure 22:**
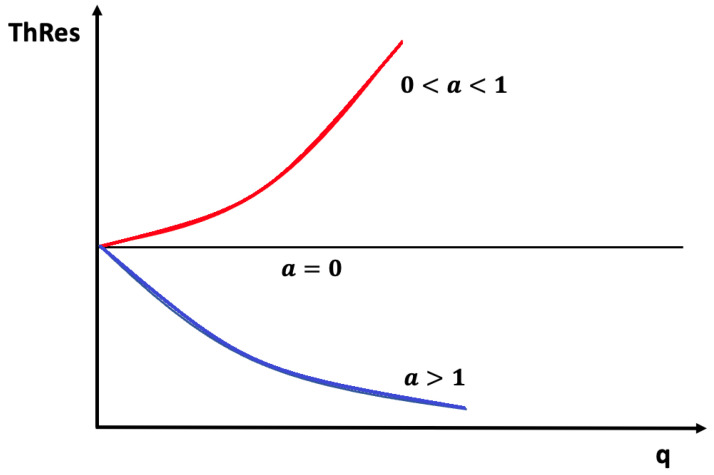
Thermal resistance as a function of q.

When b=0 and (for the sake of simplicity) the linear approximation is made into $exp\left(\frac{\alpha_{1}qL}{D}\right)-1≅\frac{\alpha_{1}qL}{D},$ (93) becomes:

(94) $$\mathrm{ThRes}=\frac{L}{\lambda_{0}}\left[1+\frac{1}{1+\frac{\beta_{1}c^{a}}{a+1}{\left(\frac{\alpha_{1}qL}{D}\right)}^{a}}\right],$$

which depends on the heat flux itself. It can be seen that depending on the value of the exponent *a*, the thermal resistance may increase, decrease, or remain constant (see Figure 22). If a>1, the thermal resistance decreases for increasing q; if 0<a<1, it increases for increasing q; if a=1, it is independent on q.

### 6.3. Charged Mobile Defects: Electrical Production of Heat Waves

**Figure 23:**
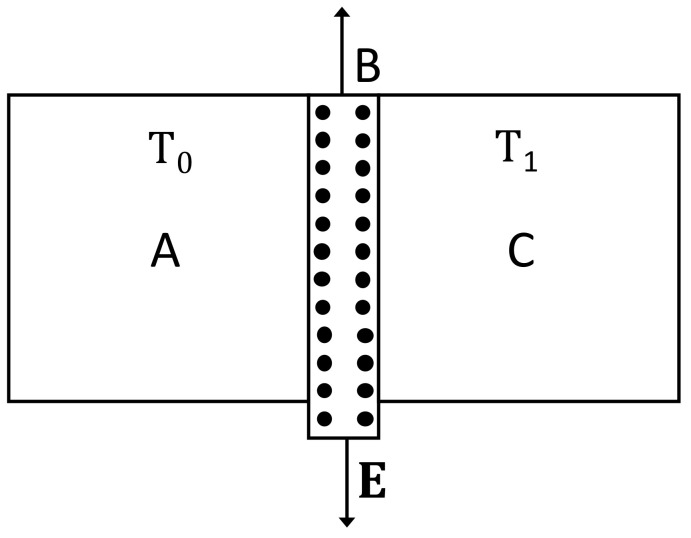
Temperature wave.

Another possible application of mobile defects would be the electrical modulation and control of the transport coefficients by means of an imposed electric field E in the case that the defects are electrically charged (see Figure 23).

Analogously, one could control the value of the transport coefficients by means of the application of a magnetic field in the case the defects have a magnetic moment. In fact, this would be analogous to the magnetoresistance effect, in which a magnetic field changes the electrical resistivity of a material. A result of the electric control of thermal conductivity would be the electrical production of temperature waves.

**Figure 24:**
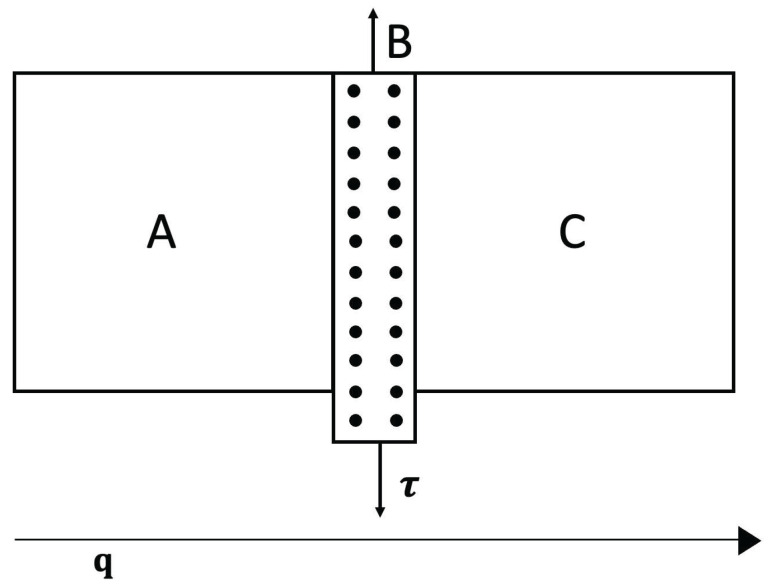
Charged defect oscillations in the layer B submitted to an oscillating electric field will produce oscillations in the heat flux from *A* to *C*.

We consider a thin layer *B* of thickness $L_{B}$ with charged mobile defects, separating a thermal reservoir *A* at a temperature $T_{0}$ from a system *C* at a lower temperature $T_{1}$. The heat flux in the steady state will be:

(95) $$q=\frac{\lambda_{B}}{L_{B}}\left[T_{0}-T_{1}\right],$$

where $\lambda_{B}$ depends on temperature *T* and on defect concentration *c*. If the defects are charged and mobile, application of an electric field along *B* will bring the positive and negative defects toward opposite borders of *B* away from the central region, thus enhancing the average thermal conductivity $\lambda_{B}$ and increasing the heat flux between *A* and *C*. If the electric field is oscillating, the heat flux across the layers will oscillate with the electric field *E* (see Figure 24). This may be useful for the production of heat waves. The equation of motion of defects will be in this case:

(96) $$\frac{dc}{dt}=D\nabla^{2}c+\nabla \cdot \left[q_{e}\alpha_{2}E\right],$$

where $\alpha_{2}E$ is the drift velocity of the respective (positive/negative) charges and $q_{e}$ their corresponding electric charge. In the case of noncharged defects, an analogous process could be made through external stress, by periodically compressing and expanding the zone *B*, thus increasing or reducing the concentration of defects.

### 6.4. Coupled Defect Waves and Temperature Waves

In this Section we consider a material with mobile defects with inertia and explore the influence of mobile defects on heat waves and their mutual coupling. Indeed, heat waves may be used as an analytical technique in material sciences but their behavior at high frequencies and small wavelengths requires going beyond the classical Fourier’ s law and the classical irreversible thermodynamics.

The system is described by the balance equation of energy and of (conserved) number of defects, respectively, yielding:

(97) $$c_{p}\left(T,c\right)\frac{dT}{dt}=-\nabla \cdot q,\;\;\frac{dc}{dt}=-\nabla \cdot J,$$

where $c_{p}$ is the specific heat at constant pressure per unit volume. To close this system of equations, equations for q and J are needed. We take for them Equations (79) and (80), with an additional relaxation term in each one:

(98) $$\tau_{1}\left(T,c\right)\frac{dq}{dt}+q=-\lambda \left(T,c\right)\nabla T,$$

(99) $$\tau_{2}\left(T,c\right)\frac{dJ}{dt}+J=-D\left(T,c\right)\nabla c+\alpha cq,$$

where $\tau_{1}$ and $\tau_{2}$ are the respective relaxation times of q and J. The relaxation time $\tau_{1}$ of q is related to the average collision time of phonons, the heat carriers inside the material, whereas $\tau_{2}$ is related to the inertia of the defects in the matrix of the material. Such inertia is related to the defect atomic mass, and describes the average time the defects need to arrive to the steady-state drift velocity under the action of the heat flux. In usual circumstances, $\tau_{1}$*is* much shorter than $\tau_{2}$. Note that in (98), it is assumed that the contribution of defects to heat transport is negligible as compared with the phonon contribution; otherwise, a term proportional to J should be added to the right-hand side of (98).

Combining (97)${}_{1}$ and (98) and assuming that the material coefficients depend on *c* and not on *T* (for the sake of simplicity of our illustration), we obtain the following:

(100) $$\frac{dc_{p}}{dc}\frac{\partial c}{\partial t}+c_{p}\frac{\partial^{2}T}{\partial t^{2}}+\frac{c_{p}}{\tau_{1}}\frac{\partial T}{\partial t}=\frac{\lambda }{\tau_{1}}\frac{\partial^{2}T}{\partial x^{2}}+\frac{\partial \tau_{1}^{-1}}{\partial c}\frac{\partial c}{\partial x}q+\frac{\partial \left(\lambda /\tau_{1}\right)}{\partial c}\frac{\partial c}{\partial x}\frac{\partial T}{\partial x}.$$

Combining (97) and (99), we have:

(101) $$\frac{\partial^{2}c}{\partial t^{2}}+\frac{1}{\tau_{2}}\frac{\partial c}{\partial t}=\frac{D}{\tau_{2}}\frac{\partial^{2}c}{\partial x^{2}}+\frac{\alpha }{\tau_{2}}cc_{p}\frac{\partial T}{\partial t}+\frac{d\left(D/\tau_{2}\right)}{dc}{\left(\frac{\partial c}{\partial x}\right)}^{2}-\frac{\alpha }{\tau_{2}}q\left(\frac{\partial c}{\partial x}\right)-cq\frac{d(\alpha /\tau_{2})}{dc}q\left(\frac{\partial c}{\partial x}\right)$$

When the material coefficients do not depend on *c* nor *T*, Equations (100) and (101) reduce to:

(102) $$c_{p}\frac{\partial^{2}T}{\partial t^{2}}+\left(c_{p}/\tau_{1}\right)\frac{\partial T}{\partial t}=\left(\lambda /\tau_{1}\right)\frac{\partial^{2}T}{\partial x^{2}},$$

(103) $$\frac{\partial^{2}c}{\partial t^{2}}+\left(1/\tau_{2}\right)\frac{\partial c}{\partial t}=\left(D/\tau_{2}\right)\frac{\partial^{2}c}{\partial x^{2}},$$

which are the telegrapher’s equations for heat waves and defect waves corresponding to the incorporation of relaxation times in (98) and (99). These equations imply that the respective high-frequency limit of the phase speed of the *T* and *c* waves are: $v_{T}^{2}=\frac{\lambda }{c_{p}\tau_{1}},v_{c}^{2}=\frac{D}{\tau_{2}}.$

Equations (100) and (101) have several nonlinear terms and second-order terms, whose mathematical treatment is complicated. Some ways of studying the influence of the several terms are: (1) considering the limit of low-frequency and long-wavelength temperature waves; (2) considering the limit of high-frequency short-wavelength waves; (3) linearizing the equations around a nonequilibrium steady state characterized by a given nonzero value of q, but a zero value of J (this state will be achieved if the system has walls which let pass heat but not defects). In this case, the reference values ${\left(\frac{\partial T}{\partial x}\right)}_{0}$ and ${\left(\frac{\partial c}{\partial x}\right)}_{0}$ are related to the reference value of $q_{0}$ through Equations (98) and (99), i.e., ${\left(\frac{\partial T}{\partial x}\right)}_{0}=-\frac{1}{\lambda }q_{0};\;\;{\left(\frac{\partial c}{\partial x}\right)}_{0}=-\frac{\alpha c}{D}q_{0}.$ We focus our interest on low-frequency waves in the presence of a steady-state heat flux $q_{0}$. In this case, Equations (100) and (101) reduce to:

(104) $$\left(\frac{c_{p}}{\tau_{1}}\right)\left(\frac{\partial T}{\partial t}\right)=\frac{d\tau_{1}^{-1}}{dc}\left(\frac{\partial c}{\partial x}\right)q_{0}+\frac{d(\lambda /\tau_{1})}{dc}{\left(\frac{\partial c}{\partial x}\right)}_{0}\frac{\partial T}{\partial x}+\frac{d(\lambda /\tau_{1})}{dc}\left(\frac{\partial c}{\partial x}\right){\left(\frac{\partial T}{\partial x}\right)}_{0}$$

and

(105) $$\left(\frac{1}{\tau_{2}}\right)\left(\frac{\partial c}{\partial t}\right)=\left(\frac{\alpha }{\tau_{2}}\right)\;c\;c_{p}\left(\frac{\partial T}{\partial t}\right)\left[\left(\frac{\alpha }{\tau_{2}}\right)+\;c\;q\frac{d(\alpha /\tau_{2})}{dc}\left(\frac{\partial c}{\partial x}\right)q_{0}.\right]$$

These may be rewritten as:

(106) $$A_{11}\left(\frac{\partial T}{\partial t}\right)=B_{11}\left(\frac{\partial T}{\partial x}\right)+B_{12}\left(\frac{\partial c}{\partial x}\right),\;\;A_{21}\left(\frac{\partial T}{\partial t}\right)+A_{22}\left(\frac{\partial c}{\partial t}\right)=B_{22}\left(\frac{\partial c}{\partial x}\right),$$

with the coefficients $A_{ij}$ and $B_{ij}$ identified by comparing (106) with (104) and (105):

$$A_{11}=\frac{c_{p}}{\tau_{1}},\;\;\;B_{11}=\left[\frac{̣(\lambda /\tau_{1})}{c̣}\right]\frac{\alpha c}{D}q_{0},\;\;\;A_{22}=\frac{1}{\tau_{2}},\;\;\;A_{21}=-\frac{\alpha \;c\;c_{p}}{\tau_{2}},\;\;\;$$

(107) $$B_{12}=\frac{d\tau_{1}^{-1}}{dc}q_{0},\;\;\;B_{22}=-\frac{d(\lambda /\tau_{1})}{dc}\frac{1}{\lambda }q_{0}.$$

Assuming plane waves:

$$T\left(x,t\right)=\hat{T}e^{\left[i(\omega t-kx)\right]}\;\mathrm{and}\;c\left(x,t\right)=\hat{c}e^{\left[i(\omega t-kx)\right]},$$

the dispersion relation from (106) leads for the phase speed $v=\frac{\omega }{k}$:

(108) $$\left|\begin{array}{cc} A_{11}v+B_{11} & B_{12}\\ A_{21}v & A_{22}v+B_{22} \end{array}\right|=0.$$

Thus:

(109) $$v=\frac{B_{12}A_{21}-A_{11}B_{22}-B_{11}A_{22}}{A_{11}A_{22}},$$

which in terms of the identifications of $A_{ij},\;B_{ij},$${\left(\frac{\partial T}{\partial x}\right)}_{0}$, and ${\left(\frac{\partial c}{\partial x}\right)}_{0}$ is:

(110) $$v=\frac{\left[\tau_{1}\alpha \;c\frac{d\tau_{1}^{-1}}{dc}+\frac{\tau_{2}}{\lambda }\frac{d(\lambda /\tau_{1})}{dc}-\frac{\alpha \;c\;\tau_{1}}{c_{p}\;D}\frac{d(\lambda /\tau_{1})}{dc}\right]q_{0}}{\left(c_{p}/\tau_{1}\tau_{2}\right)}.$$

Thus, in the low-frequency limit, there are waves of *T* and *c* propagating with a speed proportional to $q_{0}$. These waves propagate in the same direction as $q_{0}$ or in the opposite direction depending on the sign of $q_{0}$ coefficients appearing in (110).

### 6.5. Coupled Electronic Waves and Dislocation Waves

As another example of coupled wave behavior, we consider electronic waves and dislocation waves in the relaxation *n*-type Ge. When dislocations (described by a dislocation core tensor [165]) are introduced into *n*-type Ge by a deformation, they reduce the conductivity, because they act as acceptor centers which abstract electrons from the conduction band, and thus, reduce the conductivity, and scatter charges in the region surrounding each dislocation.

Confining our attention to the electronic and dislocation fields and assuming that the values of nonequilibrium perturbations of the concentration of electrons *n* and the dislocation density *a* are low, Equations (39), (44)${}_{1}$, (44)${}_{3}$, and (44)${}_{4}$ governing the electronic and dislocation fields reduce to the following linear form (see [76]):

(111) $$\rho \dot{n}+j_{k,k}^{n}=\frac{\rho n}{\tau^{+}}-ka,$$

(112) $$\tau^{n}j_{k}^{n}=\alpha_{n}a_{,k}-\rho D_{n}n_{,k}-j_{k}^{n},$$

(113) $$\dot{a}+V_{k,k}=0$$

(114) $$\tau^{a}{\dot{V}}_{k}=-D_{a}a_{,k}+\alpha_{\upsilon }n_{,k}-V_{k}.$$

Equation (111) is (39)${}_{1}$ with a coupling to the dislocation density *a*; Equation (112) is a particular case of (44)${}_{1}$; Equation (113) is (44)${}_{3}$ with $A_{ij}\equiv 0$; and Equation (114) generalizes the diffusive form of the dislocation flow $V_{k}=-Da_{,k}$ (44)${}_{4}$, by taking into account the relaxational term $\tau^{a}{\dot{V}}_{k}$, and the coupling term $\alpha_{\upsilon }n_{,k}$. Then, z the recombination function $g^{n}$ (see (48)) should depend on the dislocation field; therefore, it has the form of (111). $\tau^{+}$ denotes the life time of electrons, $\tau^{n}$ is the relaxation time of electrons, $D_{n}$ and $D_{a}$ are the diffusion coefficients of electrons and dislocations, respectively, $\tau^{a}$ denotes the relaxation time of dislocations, *k* is the recombination constant due to dislocations, and $\alpha_{n}$, $\alpha_{\upsilon }$ are coupling constants reflecting cross-kinetic effects during electronic-dislocation interactions. From now on, the superimposed dot denotes partial time derivative. Equation (113) shows “*the number*” of dislocations (there is no production of them) so that the dislocation life-time is assumed as infinitely long.

From the set (111)–(114), there result the following coupled equations for the considered waves:

(115) $$D_{n}n_{,kk}-\left(1-\frac{\tau^{n}}{\tau^{+}}\right)\dot{n}-\tau^{n}\ddot{n}+\frac{n}{\tau^{+}}-\frac{\alpha_{n}}{\rho }a_{,kk}-\frac{k\tau^{n}}{\rho }\dot{a}-\frac{k}{\rho }a=0,$$

(116) $$D_{a}a_{,kk}-\dot{a}-\tau^{a}\ddot{a}-\alpha_{\upsilon }n_{,kk}=0.$$

To find the propagation conditions and dispersion of the electronic-dislocation (ED) waves, we consider $\tau^{+}=\tau^{n}$ [76] and solutions of the form:

(117) $$n=\hat{n}e^{ik(x-vt)},\;a=\hat{a}e^{ik(x-vt)}.$$

We obtain from (115) and (116):

$$\left(-k^{2}c_{n}^{2}+k^{2}v^{2}+\frac{1}{{\left(\tau^{n}\right)}^{2}}\right)n+\left(m_{1}k^{2}+m_{2}ikv-m_{3}\right)a=0$$

(118) $$m_{4}k^{2}n+\left(-k^{2}c_{a}^{2}+\frac{ikv}{\tau^{a}}+k^{2}v^{2}\right)a=0,$$

where the following notation has been used:

(119) $$c_{n}^{2}=\frac{D_{n}}{\tau^{n}},\;\;c_{a}^{2}=\frac{D_{a}}{\tau^{a}},\;\;m_{1}=\frac{\alpha_{n}}{\rho \tau^{n}},\;\;m_{2}=\frac{k}{\rho },\;\;m_{3}=\frac{k}{\rho \tau^{n}},\;\;m_{4}=\frac{\alpha_{v}}{\tau^{a}}.$$

System (118) has nontrivial solutions only if its determinant vanishes, namely:

(120) $$D=\left|\begin{array}{cc} -k^{2}c_{n}^{2}+k^{2}v^{2}+\frac{1}{{\left(\tau^{n}\right)}^{2}} & m_{1}k^{2}+m_{2}ikv-m_{3}\\ m_{4}k^{2} & -k^{2}c_{a}^{2}+\frac{ikv}{\tau^{a}}+k^{2}v^{2} \end{array}\right|=0.$$

Expression (120) yields the dispersion relation of the considered waves.

Developing D we obtain velocities concerning four possible modes [76]:

(121) $$v_{\left(1),(2\right)}=\sqrt{r_{1}\pm \sqrt{r_{1}^{2}-r_{2}}},\;v_{\left(3\right)}=0\;\mathrm{and}\;v_{\left(4\right)}=\sqrt{c_{n}^{2}+\frac{1}{{\left(k\tau^{n}\right)}^{2}}}+m_{2}m_{4}\tau^{a},$$

where

(122) $$2r_{1}=c_{n}^{2}+c_{a}^{2}+\frac{1}{{\left(k\tau^{n}\right)}^{2}},\;r_{2}=c_{n}^{2}c_{a}^{2}-m_{1}m_{4}+\frac{1}{k^{2}}\left(m_{3}m_{4}+\frac{c_{a}^{2}}{{\left(\tau^{n}\right)}^{2}}\right).$$

**Figure 25:**
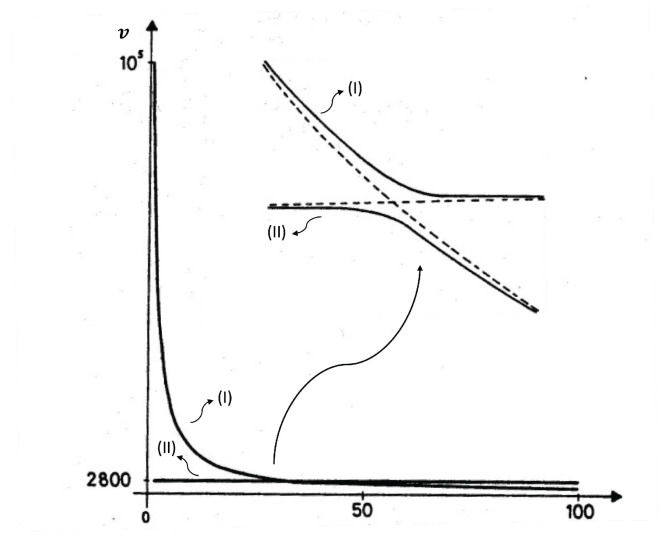
Dispersion of electronic-dislocation (ED) waves: (I)—the electronic branch, (II)—the dislocation branch, after [76].

Simple analysis of (121)–(122) shows that there are two groups of waves propagating in the body: the ED mode $v_{\left(1\right)}$ (the electronic branch, indicated by (I)), the ED mode $v_{\left(2\right)}$ (the dislocation branch, indicated by (II)), and the modified electronic mode $v_{\left(4\right)}$, whose velocity includes the influence of the dislocation field. The values of $v_{\left(4\right)}$ are higher than the values of the pure electronic modes (cf. [76], where they have been called the pure plasmonic modes in the bulk). In [76] the dispersion curves for *n*-type Ge plotted in Figure 25 have been obtained estimating the coefficients in (115) and (116), using (119), see Table 1.

From Figure 25, the results show that there occurs a resonance in the electronic-dislocation system in Ge for ω = 10${}^{5}$ s${}^{-1}$, velocity v=2800 m/s and wave-length λ=0.167 m. According to the modified electronic wave, the contribution of coupling coming from the dislocation field is in principle, negligible, being $m_{2}m_{4}\tau^{a}$ = 0.025 m${}^{2}$/s${}^{2}$ (in comparison with $c_{n}^{2}$ in (121)${}_{3}$).

### 6.6. Temperature Waves

Another particularly interesting case are temperature waves, which have been considered in a simplified way in Section 6.4 coupled with defect waves. We consider heat Equation (79), rewritten for convenience:

$$\tau^{q}{\dot{q}}_{i}=\gamma_{ijk}^{1}\varepsilon_{jk}-\gamma_{ij}^{2}E_{j}+\gamma_{ijk}^{3}\alpha_{jk}+\gamma_{ij}^{4}j_{j}^{n}+\gamma_{ij}^{5}j_{j}^{p}$$

(123) $$-q_{i}-\gamma_{ij}^{7}\theta_{,j}+\gamma_{ijkl}^{8}\alpha_{jk,l}+\gamma_{ij}^{9}N_{,j}+\gamma_{ij}^{10}P_{,j},$$

where $\;\gamma^{s}=\tau^{q}\chi^{s}\left(s=1,2,\ldots ,5,7,8\ldots ,10\right),\;\;\delta_{ij}={\left(\tau^{q}\right)}^{-1}\chi_{ij}^{6}.$

When the coefficients $\gamma^{s}$ are negligible, Equation (123) takes the form of anisotropic Maxwell–Cattaneo–Vernotte heat equation $\tau^{q}{\dot{q}}_{i}+q_{i}=-\gamma_{ij}^{7}\theta_{,j}$, plus coupling to the electric field, electric currents $j^{n}$ and $j^{p}$, and dislocation field. Using the expression F=U−TS, its time derivative, the internal energy balance (37), the state laws (49), the definitions of affinities (50), Equations (123) and (59)${}_{2}$, and linearizing around the equilibrium state, we have:

$$\tau^{q}\rho_{0}T_{0}\left(\frac{\lambda_{ij}^{\theta }}{\rho_{0}}{\ddot{\varepsilon }}_{ij}+\lambda_{i}^{\theta }{\ddot{E}}_{i}+\frac{c}{T_{0}}\ddot{\theta }-\frac{\alpha_{ij}^{a\theta }}{\rho_{0}}{\ddot{\alpha }}_{ij}+\lambda^{\theta N}\ddot{N}+\lambda^{\theta P}\ddot{P}\right)=$$

$$-\tau^{q}\rho_{0}T_{0}\left(\frac{\lambda_{ij}^{\theta }}{\rho_{0}}{\dot{\varepsilon }}_{ij}+\lambda_{i}^{\theta }{\dot{E}}_{i}+\frac{c}{T_{0}}\dot{\theta }-\frac{\alpha_{ij}^{a\theta }}{\rho_{0}}{\dot{\alpha }}_{ij}+\lambda^{\theta N}\dot{N}+\lambda^{\theta P}\dot{P}\right)-\gamma_{ijk}^{1}\varepsilon_{jk,i}+$$

(124) $$\gamma_{ij}^{2}E_{j,i}-\gamma_{ijk}^{3}\alpha_{jk,i}-\gamma_{ij}^{4}j_{j,i}^{n}-\gamma_{ij}^{5}j_{j,i}^{p}+\gamma_{ij}^{7}\theta_{,ji}-\gamma_{ijkl}^{8}\alpha_{jk,li}-\gamma_{ij}^{9}N_{j,i}-\gamma_{ij}^{10}P_{j,i}.$$

Introducing $k_{ij}=\frac{T_{0}}{\rho_{0}c}\lambda_{ij}^{\theta \varepsilon },\;\varphi_{i}^{1}=\frac{T_{0}}{c}\lambda_{i}^{\theta },\;\varphi^{2}=\frac{T_{0}}{c}\lambda^{N},\;\;\varphi^{3}=\frac{T_{0}}{c}\lambda^{P},\;\eta_{ij}=\frac{T_{0}}{\rho_{0}c}\lambda_{ij}^{a\theta },\nu^{s}=\frac{\gamma^{s}}{\rho_{0}c}s=1,2,\ldots ,5,7,8\ldots ,10)$, we obtain a generalized telegraph heat equation leading to finite speeds of propagation of thermal disturbances:

$$\tau^{q}\ddot{\theta }+\dot{\theta }=\gamma_{ij}^{7}\theta_{,ji}-k_{ij}\left(\tau^{q}{\ddot{\varepsilon }}_{ij}+{\dot{\varepsilon }}_{ij}\right)+\varphi_{i}^{1}\left(\tau^{q}{\ddot{E}}_{i}+{\dot{E}}_{i}\right)+\varphi^{2}\left(\tau^{q}\ddot{N}+\dot{N}\right)+$$

$$\varphi^{3}\left(\tau^{q}\ddot{P}+\dot{P}\right)+\eta_{ij}\left(\tau^{q}{\ddot{\alpha }}_{ij}+{\dot{\alpha }}_{ij}\right)-\gamma_{ijk}^{1}\varepsilon_{jk,i}+\gamma_{ij}^{2}E_{j,i}-\gamma_{ijk}^{3}\alpha_{jk,i}$$

(125) $$-\gamma_{ij}^{4}j_{j,i}^{n}-\gamma_{ij}^{5}j_{j,i}^{p}+\gamma_{ij}^{7}\theta_{,ji}-\gamma_{ijkl}^{8}\alpha_{jk,li}-\gamma_{ij}^{9}N_{j,i}-\gamma_{ij}^{10}P_{j,i}.$$

When the semiconductors are intrinsic, without defects or deformations, the effects of the electric field are negligible and the superimposed dot denotes partial time derivative, and Equation (125) reduces to the telegraph equation:

(126) $$\tau^{q}\ddot{\theta }+\dot{\theta }=\gamma_{ij}^{7}\theta_{,ji},$$

where $\gamma_{ij}^{7}=\frac{\lambda }{\rho \;c_{p}}$. In (125), many other other couplings may be considered.

## 7. Summary and Outlook

We have studied a thermodynamic framework, paying special attention to the control of the transport coefficients of the system (heat conductivity and thermoelectric coefficients). Such coefficients depend on T and the reference material and also on the defects, applied stress, microstructure, and inhomogeneities of the material. This yields nonlinear effects, allowing to regulate the several fluxes with precision.

First, as a motivation and overview of the context, we have referred to defect engineering [80,81,82,83,84,85,86,87,88,89,90,91], dislocation engineering [1,2,3,4,5,6,7], stress engineering [92,93,94,95,96,97], phonon engineering [98,99,100,101,102], and nanoengineering, to see the several aims of these relatively new engineering techniques on which much current activity is focused. Second, we have examined the nonequilibrium thermodynamic context; using procedures of rational extended thermodynamics, we have derived in the anisotropic case the constitutive equations and the rate equations for charges and heat fluxes, obtaining a balanced system of equations to describe extrinsic semiconductors and superlattices with defects of dislocations. The models for these media may have relevance in several fundamental technological fields: applied computer science, integrated circuits VLSI (Very Large Scale Integration), LEDs and lasers, electronic microscopy, nanotechnology, photovoltaic cells, and thermoelectric energy conversion.

Several different thermodynamic formalisms could be used, as for instance thermodynamics with internal variables [75,105], as well as extended thermodynamics [55,56,200]. Thus, among the several equations in this paper, we have paid special attention to the evolution equation for defects, when the system is submitted to electric fields as temperature gradients. Because of the influence of defects, stress, and structures on the transport coefficients, the action of external fields on these components of the system has a strongly nonlinear effect. In particular, the dependence of the electrical conductivity on an applied electric field in some kinds of polarized systems or in systems with charged defects (see [46,201,202,203,204,205]) may open new ways to control the heat flux in an easily tunable way. We have considered a number of possible new applications suggested by the present formalism.

## Figures and Tables

**Table 1:** The estimated coefficients, after [76].

Coefficient	Measure Unit	Value	Name
ρ	Kg m${}^{-3}$	$5.3\times 10^{3}$	mass density [193,198]
$D_{n}$	m${}^{2}$ s${}^{-1}$	$10^{-2}$	electron diffusion coefficient [193,198]
$D_{a}$	m${}^{2}$ s${}^{-1}$	$2.43\times 10^{-2}$	dislocation diffusion coefficient (119)
$\tau^{n}$	s	<$10^{-5}$	electron relaxation time [198]
$\tau^{a}$	s	$0.3\times 10^{-8}$	dislocation relaxation time [160]
$\alpha_{n}$	C m s${}^{-1}$	<1.8	cross-effects function, estimated [161,199]
$\alpha_{v}$	Kg C${}^{-1}$ s${}^{-1}$	<70	cross-effects function, estimated [161,199]
κ	C m${}^{-1}$ s${}^{-1}$	<$1.9\times 10^{-4}$	recombination constant, estimated [161]
$c_{n}$	m s${}^{-1}$	$10^{3}$	$\sqrt{D_{n}/\tau^{n}}$ (119)
$c_{a}$	m s${}^{-1}$	2846	$\sqrt{D_{a}/\tau^{a}}$ [160]

## Data Availability

The authors agree with the availability of their research data.
